# Design, Synthesis, and Evaluation of Homochiral Peptides Containing Arginine and Histidine as Molecular Transporters

**DOI:** 10.3390/molecules23071590

**Published:** 2018-06-29

**Authors:** Naglaa Salem El-Sayed, Taryn Miyake, Amir Nasrolahi Shirazi, Shang Eun Park, Jimmy Clark, Stephani Buchholz, Keykavous Parang, Rakesh Tiwari

**Affiliations:** 1 Center for Targeted Drug Delivery, Department of Biomedical and Pharmaceutical Sciences, Chapman University School of Pharmacy, Harry and Diane Rinker Health Science Campus, Irvine, CA 92618, USA; nsibrahim18@gmail.com (N.S.E.-S.); miyak106@mail.chapman.edu (T.M.); nasrolahishirazi@gmail.com (A.N.S.); park327@mail.chapman.edu (S.E.P.); clark217@mail.chapman.edu (J.C.); buchh101@mail.chapman.edu (S.B.); parang@chapman.edu (K.P.); 2 Cellulose and Paper Department, National Research Center, Dokki, Cairo 12622, Egypt

**Keywords:** anticancer drugs, fatty acyl peptides, histidine, phosphopeptides, molecular transporters, cytotoxicity

## Abstract

Linear (HR)_n_ and cyclic [HR]_n_ peptides (n = 4,5) containing alternate arginine and histidine residues were synthesized. The peptides showed 0–15% cytotoxicity at 5–100 µM in human ovarian adenocarcinoma (SK-OV-3) cells while they exhibited 0–12% toxicity in human leukemia cancer cell line (CCRF-CEM). Among all peptides, cyclic [HR]_4_ peptide was able to improve the delivery of a cell impermeable fluorescence-labeled phosphopeptide by two-fold. Fatty acids of different alkyl chain length were attached at the *N*-terminal of the linear peptide (HR)_4_ to improve the molecular transporter property. Addition of fatty acyl chains was expected to help with the permeation of the peptides through the cell membrane. Thus, we synthesized seven fatty acyl derivatives of the linear (HR)_4_ peptide. The peptides were synthesized using Fmoc/*t*Bu solid phase peptide chemistry, purified by reverse-phase high-performance liquid chromatography (RP-HPLC) and characterized by matrix-assisted laser desorption/ionization (MALDI) spectrometry. The fatty acyl peptides containing C_8_, C_12_, C_14_, and C_18_ alkyl chain did not show cytotoxicity on SK-OV-3 or CCRF-CEM cell lines up to 50 µM concentration; however, at higher concentration (100 µM), they showed mild cytotoxicity. For example, C_16_-(HR)_4_ was also found to reduce the proliferation of SK-OV-3 cells by 11% at 50 µM and C_20_-(HR)_4_ showed mild toxicity at 10 µM, reducing the proliferation of SK-OV-3 cells by 21%. Increase in the length of alkyl chain showed cytotoxicity to the cell lines. C_20_-(HR)_4_ peptide showed better efficiency in translocation of F′-GpYEEI to SK-OV-3 than the phosphopeptide alone. Further investigation of C_20_-(HR)_4_ peptide efficacy showed that the peptide could deliver doxorubicin and epirubicin into SK-OV-3 and also improved the drug antiproliferative ability. These studies provided insights into understanding the structural requirements for optimal cellular delivery of the fatty acyl-(HR)_4_ peptide conjugates.

## 1. Introduction

Cell-penetrating peptides (CPPs) also known as protein transduction domains (PTDs), are a group of short peptides with up to 25–30 amino acids that are capable of penetrating the cell membrane. CPPs are characterized by their high loading capacity, high transduction efficiency and rapid transduction rate [1,2]. Therefore, they are considered as good candidates for translocation of bioactive macromolecules, such as proteins, nucleic acids, peptides, inorganic particles and liposomes into cells [3,4,5,6].

CPPs are classified according to their physicochemical nature into amphipathic, hydrophobic or cationic peptides. Other classification is based on the origin of CPPs, dividing them into protein-derived CPPs, for example, TAT peptide and penetratin, model peptides as a MAP, KLAL, polyarginine, and finally designed peptides or chimeric peptides, which consist of functional domains of target proteins [7,8]. 

CPPs differ from one to another in the routes of entry and intracellular transport mechanism for the therapeutic agents [9]. Two major routes for CPPs cellular internalization have been proposed: direct membrane translocation via pore-opening mechanism and endocytosis-mediated pathway, which divides into clathrin-dependent endocytosis, actin-dependent and caveolae-dependent micropinocytosis pathways [10]. The endosomal entrapment, potential cytotoxicity, immunogenicity and the lack of selectivity are the main obstacles that hinder the wide use of CPP’s in the fields of imaging, drug delivery, and treatment [11]. 

Moreover, targeting the tumor tissue could be achieved through active targeting using specific tumor-associated biomarkers. However, active tumor triggering approaches have some difficulties because of the heterogeneity of the tumor tissues and the potential to develop resistance [12]. Therefore, the development of alternate generic targeting strategies using a global feature of various cancers, such as hypoxia [13], vasculature [14], or tumor acidic pH [15], are subjects of major interest. The diverse properties of naturally occurring amino acids permit their use for developing multifunctional CPPs that vary in their physicochemical properties including their degree of amphiphilicity, pKa, as well as their biological activities. 

Among these amino acids, histidine (abbreviated as His or H) is an essential amino acid with a protonable imidazolyl group, which is required for many enzymatic activities [16]. Furthermore, several reports emphasized the impact of incorporation of histidine amino acid into a peptide sequence that can be used for various applications. For example, Gaspar et al. reported the successful isolation and recovery of minicircle DNA biopharmaceuticals by using a histidine-based peptide of sequence (C_2_H_6_)/zinc ions complex [16]. Liu et al. reported that histidine-containing nona arginine (HR_9_) combined with semiconductor quantum dots (QDs) are efficient, non-toxic drug delivery systems [17]. Li et al. demonstrated that co-grafting of arginine and histidine amino acids onto the poly(ω-aminohexyl methacrylamide) (PAHMAA) polymer improved the polymer serum-compatibility, gene transfection efficacy and decreased its cytotoxicity [18]. Replacement of all basic amino acids in the antimicrobial peptide ARK24 and AKK24 by histidines yielded the histidine-rich peptides AHH24:1 and AHH24:2, respectively and the addition of Zn^2+^ restored the antimicrobial activity of the AHH peptides. Bacalum et al. reported that the substitution of tryptophan by histidine in the antimicrobial peptide sequence R_2_W_2_RW_2_R_2_ modulated the antimicrobial activity [19]. 

In our previous studies, we explored the combination of attaching different hydrophobic amino acids and charged residues with or without fatty acids to get homochiral CPPs as molecular transporters named [WR]_4_ and [WR]_5_ [2,20,21,22,23,24]. Also, a further effort led to the development of new series of peptides named [CR]_4_ and [CR]_5_ which contains cysteine and arginine residues that showed an efficient molecular transporter’s property for a diverse group of bioactive molecules [25]. In the frame work of developing unique CPPs to explore their application of molecular transporter properties and the targeting of cargos to cancer cells, we aimed to develop novel CPPs with alternating arginine and histidine amino acids to evaluate their ability to translocate various biomolecular cargoes of different molecular size and hydrophobic nature to the cancer cells. We hypothesized that the hydrophilic positively-charged side chain of arginine could interact with the negatively-charged phospholipid of the cell membrane. Moreover, the hydrophilic nature and pKa value of histidine imidazolyl ring (~6.0) could affect the transport of the cargos based on pH of cancerous cells [26]. Also, the impact of attaching fatty acid on modulating the activity and cytotoxicity of [HR]_4_ peptides was investigated. The cytotoxicity and transporting ability for many bioactive molecules by the synthesized peptides were studied at different peptides concentrations.

## 2. Result and Discussion

### 2.1. Chemistry

#### 2.1.1. Synthesis of Linear and Cyclic Peptides 

**Scheme 1:**
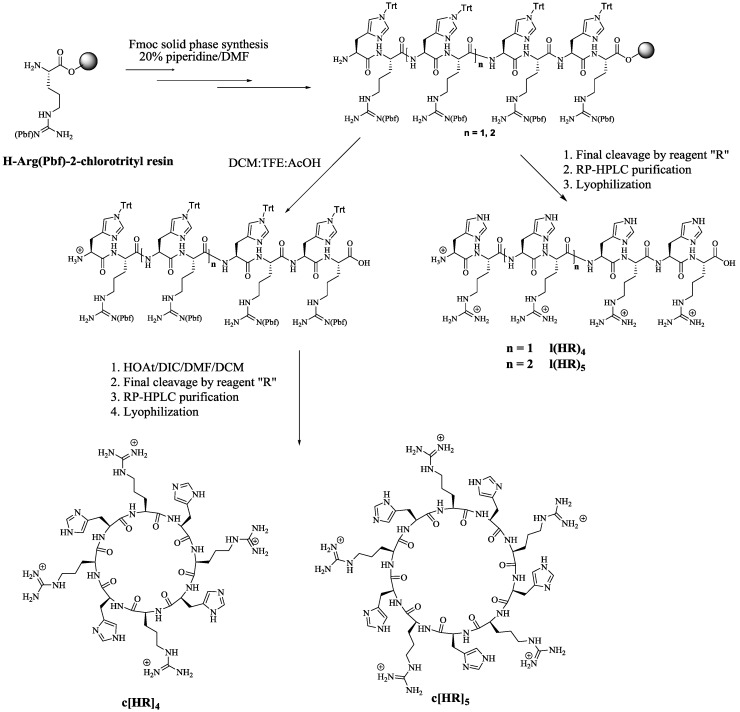
Synthesis of l(HR)_4_, l(HR)_5,_ c[HR]_4,_ and c[HR]_5_.

Four homochiral amphipathic peptides of sequence linear (HR)_4_, linear (HR)_5_, cyclic [HR]_4_ and cyclic [HR]_5_ were synthesized by standard Fmoc solid-phase peptide synthesis (SPPS) on 2-chlorotrityl resin as solid support. Parentheses’ ( ) represent linear (l) peptide where as square brackets’ [ ] represent cyclic [c] peptide. All peptides were purified by preparative RP-HPLC and analyzed by MALDI-TOF/TOF mass spectroscopy (see Supplementary Materials). Scheme 1 depicts the synthesis of the peptides. The protected amino acids were assembled on H-Arg(Pbf)-2-chlorotrityl resin. Cleavage of the resin in the presence of DCM:TFE:AcOH followed by cyclization generated cyclic peptides [HR]_4_ and [HR]_5_. Complete deprotection of the resin and the protecting groups in the presence of reagent R (trifluoroacetic acid (TFA)/thioanisole/anisole/1,2-ethanedithiol (EDT) (90:5:2:3, *v*/*v*/*v*/*v*) afforded linear peptides l(HR)_4_ and l(HR)_5_. 

#### 2.1.2. Synthesis of Fatty Acyl Linear (HR)_4_ Peptides

**Scheme 2:**
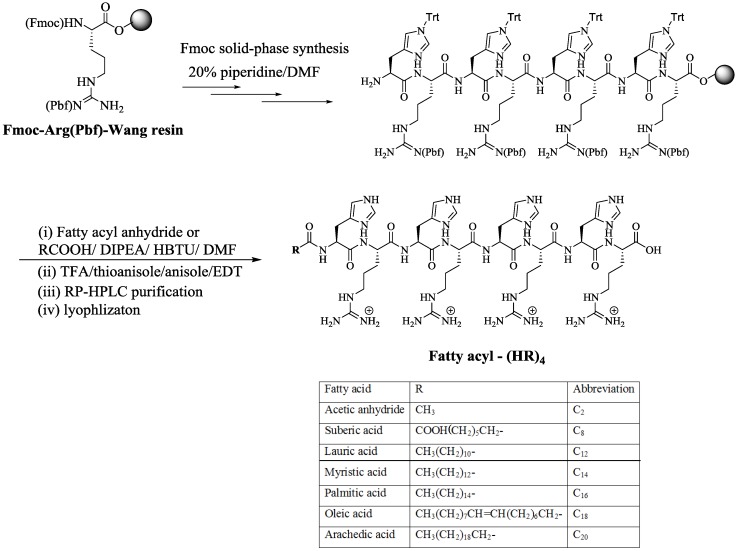
Synthesis of fatty acyl conjugates of linear (HR)_4_ peptides.

**Figure 1:**
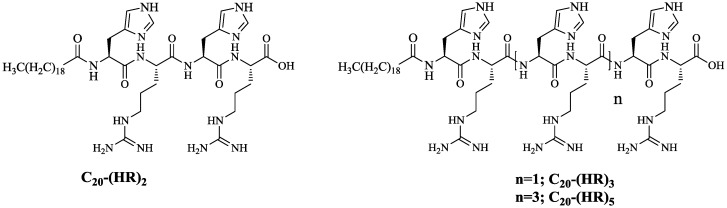
Chemical structures of C_20_-(HR)_2_, C_20_-(HR)_3_, and C_20_-(HR)_5_.

It was expected that the attachment of fatty acids or hydrophobic residues to a cationic CPPs will increase the peptide′s hydrophobicity. Hydrophobicity enhances the interactions with the cell membrane phospholipids leading to increased cellular uptake, and/or modulating both activity and selectivity [25,27,28,29]. Thus, a library of fatty acyl-(HR)_4_ peptides were synthesized using Wang resin as the solid support as shown in Scheme 2. After peptide assembly on the solid-phase resin, the peptide underwent reaction with a fatty acyl anhydride or other selected fatty acid namely suberic acid (C_8_), lauric acid (C_12_), myristic acid (C_14_), palmitic acid (C_16_), oleic acid (C_18_), and arachidic acid (C_20_) using HBTU as activating agent and DIPEA as a base. C_20_-(HR)_2_, C_20_-(HR)_3_, and C_20_-(HR)_5_, (Figure 1) were also synthesized using a similar procedure to correlate the peptide cellular transporter activities with the number of amino acids residues. 

### 2.2. Cell Assay

#### 2.2.1. Peptide Cytotoxicity

**Figure 2:**
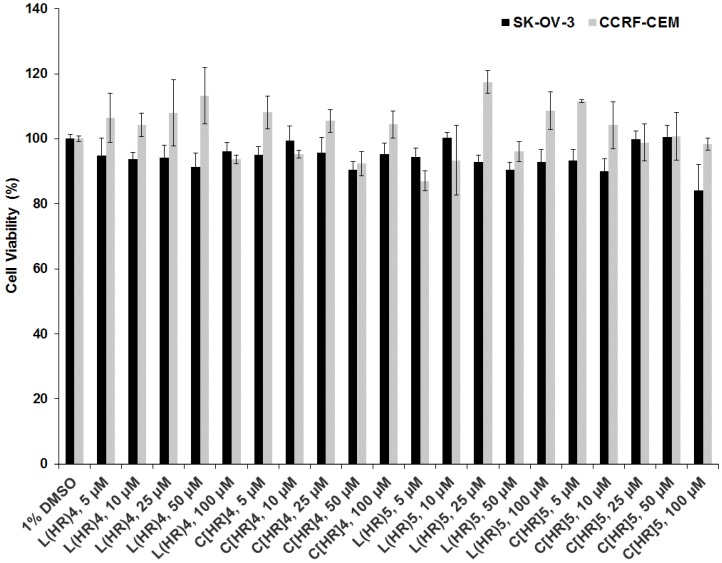
Cytotoxicity study for l(HR)_4_, l(HR)_5_, c[HR]_4_, and c[HR]_5_ at 5, 10, 25, 50, and 100 µM in SK-OV-3 and CCRF-CEM cell lines after 24 h incubation.

The cytotoxicity of l(HR)_4_, l(HR)_5_, c[HR]_4_, and c[HR]_5_ was evaluated in human ovarian (SK-OV-3) and leukemia (CCRF-CEM) cancer cells at different peptides concentrations (5, 10, 25, 50, and 100 µM) after incubation for 24 h (Figure 2). The peptide solutions had a final concentration of 1% DMSO in the assay. The peptides showed 5–10% toxicity at 5–100 µM peptide concentration in SK-OV-3 and 0–13% in CCRF-CEM at the same concentrations as compared to negative control (1% DMSO). From the cytotoxicity studies, it is clear that no significant difference was observed in the peptides cytotoxicity between linear and cyclic arginine/histidine-containing peptides and the number of amino acids did not demonstrate a detectable difference in the toxicity profile of the four peptides at all the investigated concentrations. 

**Figure 3:**
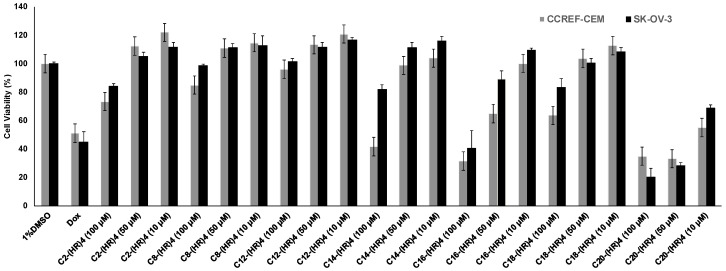
Cytotoxicity study for the fatty acyl-(HR)_4_ derivatives at concentrations of 10, 50 and 100 µM after 72 h incubation with CCRF-CEM and SK-OV-3 cells.

However, the change in the cytotoxicity profile was observed upon the attachment of fatty acids of different chain length and degree of hydrophobicity to l(HR)_4_ peptide. l(HR)_4_ conjugated with acetyl (C_2_), suberic (C_8_), lauroyl (C_12_), myristoyl (C_14_), and oleoyl (C_18_) fatty acids substituent showed 0–2% cytotoxicity on CCRF-CEM and 0% on SK-OV-3 at 10 and 50 µM peptide concentration as compared to negative (1% DMSO) and positive (Doxorubicin (Dox, 10 µM)) control. Increasing the concentration to 100 µM, caused a reduction in the cell viability for l(HR)_4_ conjugated with C_2_ by 16%, C_8_ by 15%, C_12_ by 0%, C_14_ by 18%, and C_18_ by 27%, respectively in SK-OV-3. The viability in CCRF-CEM cell line was reduced by 27–60% when the cells were treated with peptides at 100 µM. Although the palmitoyl conjugate, C_16_-(HR)_4_, had 0% cytotoxic effect on both the cell lines at 10 µM and the cell viability was reduced by 35–70% at peptide concentrations of 50 and 100 µM, respectively. The arachidyl peptide conjugate, C_20_-(HR)_4_, was toxic at all concentrations, where the cell viability was significantly reduced by (45–66%) in CCRF-CEM cells and by (31–80%) in SK-OV-3 cells, as illustrated in (Figure 3). Cancer cells proliferate and produce lactic acid and result in acidic pH (~6.5). We assume that the observed cytotoxicity of peptide was due to an increase of hydrophobicity of the fatty acyl peptide (C_20_-(HR)_4_) and cellular permeability as compared to (HR)_4_ peptide. The pH of the media for both cancer cell lines after 72 h of treatment for l(HR)_4_ peptide was found to be acidic (pH ~ 6.5) in comparison to the weak basic pH (~7.5) for C_20_-(HR)_4_ treated cells. The untreated control cells were found to have a pH of 6.5, showing 100% proliferation in the cancer cells. (HR)_4_ is protonated at an acidic pH of the medium that results in membrane ionic interactions that did not improve permeability due to the lack of hydrophobicity to interact with the lipid residues in the membrane. On the other hand, fatty acyl compounds, such as C_16_-(HR)_4_ and C_20_-(HR)_4_ analogs, resulted in the cytotoxicity. The pH of cellular media was found to be ~7.5 for these compounds. At this pH, the majority of histidine residues are more deprotonated and the compound is more hydrophobic. Furthermore, C_20_ acyl chain contributes to increased hydrophobicity, and more cellular permeability occurs due to the enhanced hydrophobicity. The enhanced hydrophobicity at higher pH is due to a higher ratio of deprotonation in histidine residues in the series of fatty acyl-(HR)_4_ peptides that lead to enhanced cellular permeability. Thus, a small difference in pH (6.5–7.5) has a significant effect on the proliferation of cancer cells. Therefore, fatty acylation significantly changed the property of l(HR)_4_ peptides.

#### 2.2.2. Molecular Transporting Efficiency of the Peptides

The synthesized conjugated peptides were evaluated for their ability to mediate the cellular internalization for fluorescence-labeled phosphopeptide containing the sequence F′-GpYEEI (where F′ represents carboxyfluorescein and pY represents phosphotyrosine amino acid). Phosphopeptides are considered as reagent probes that mimic phosphoproteins involved in signal transduction. They are usually employed in studying the mechanism of phosphoprotein–protein, protein-ligand interactions, the determination of phosphatases enzyme’s specificity to substrates, and the identification of phosphotyrosine receptors binding domains (PTB) [21,30]. The presence of negatively-charged phosphate groups in addition to the hydrophobic nature of phosphopeptides limited their ability to bypass the cell membrane and reduce their cellular uptake. Consequently, several approaches have been suggested to promote their transportation through the cell membrane into the cell cytoplasm [21,26]. 

**Figure 4:**
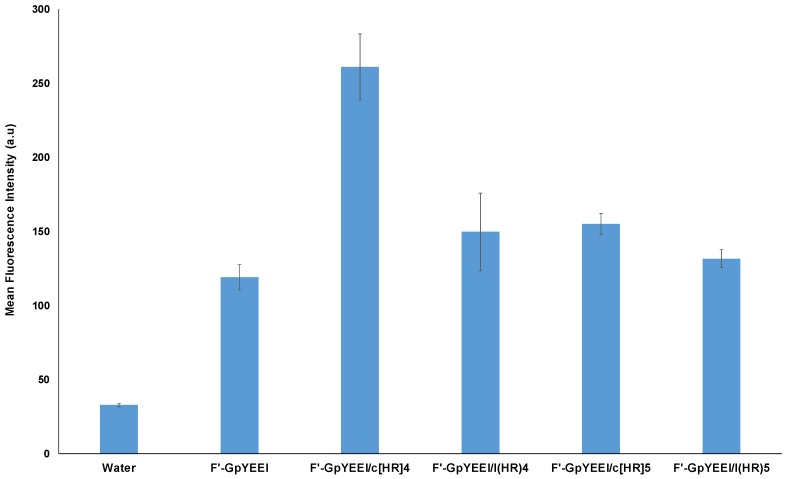
Cellular uptake study of F′-GpYEEI phosphopeptide at 5 µM by c[HR]_4_, c[HR]_5_, l(HR)_4_, and l(HR)_5_ at 50 µM peptide concentration in SK-OV-3 cancer cell lines.

Fluorescently labeled phosphopeptide F′-GpYEEI at a concentration of 5 µM was incubated with the linear and cyclic peptides (l(HR)_4_, l(HR)_5_, c[HR]_4_, and c[HR]_5_) at a concentration of 50 µM for 2 h. The cellular internalization for F′-GpYEEI by c[HR]_5_, l(HR)_4_, and l(HR)_5_ did not differ significantly from that of the phosphopeptide alone. However, the cellular uptake of F′-GpYEEI was increased by two folds when loaded with c[HR]_4_. These data indicate that only cyclic [HR]_4_ was able to promote the delivery of F′-GpYEEI (Figure 4).

**Figure 5:**
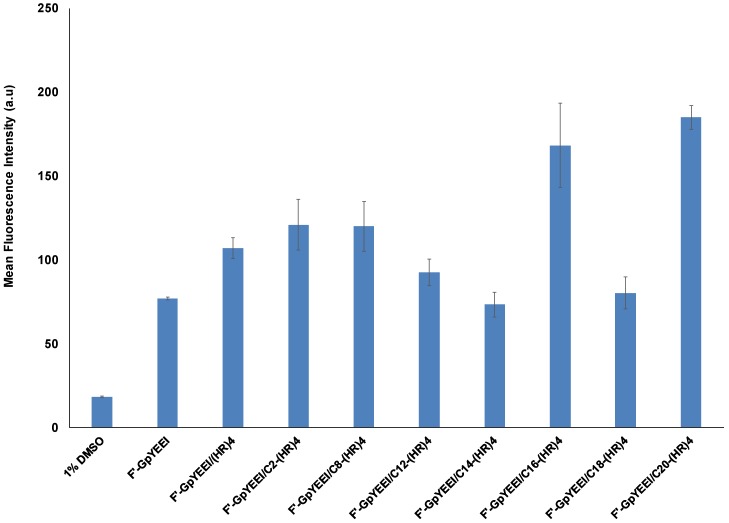
Cellular uptake study of F′-GpYEEI (5 µM) by fatty acyl-(HR)_4_ derivatives at 50 µM in SK-OV-3 cancer cell lines.

**Figure 6:**
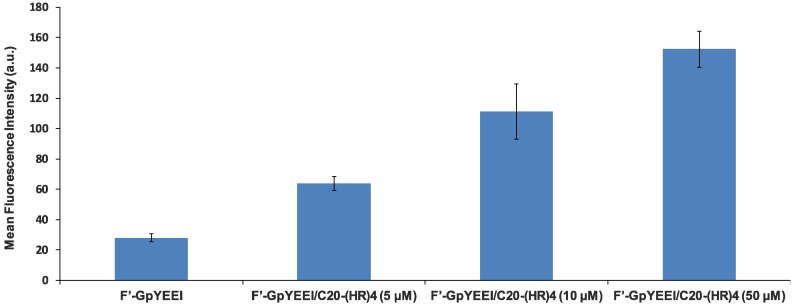
Cellular uptake studies F′-GpYEEI (5 µM) in the presence of C_20_-(HR)_4_ in SK-OV-3 cell lines at a concentration of 5, 10 and 50 µM of C_20_-(HR)_4_.

The four alternate H and R residues showed ability in delivering phosphopeptide (F′-GpYEEI) inside the SK-OV-3 cells. Therefore, we investigated the impact of attachment of fatty acyl chain with different hydrophobic alkyl chains to l(HR)_4_ in modulating the cellular uptake using fatty acylation strategy [27]. The conjugation of fatty acyl chain to l(HR)_4_ peptide affected the molecular transportation ability of F′-GpYEEI peptide. When F′-GpYEEI was incubated with the fatty acyl-(HR)_4_ peptides at a concentration of 50 µM for 2 h, it was found that C_16_-(HR)_4_ and C_20_-(HR)_4_ were the most effective in promoting the cellular uptake. They improved the cellular uptake of F′-GpYEEI by 1.5 and 2.0 folds, respectively, as compared to the free form of F′-GpYEEI. Fatty acyl (HR)_4_ peptides containing acetyl or suberoyl moieties increased the uptake of F′-GpYEEI by 10%, while (HR)_4_ peptides with the lauroyl, myristoyl, and oleoyl moieties slightly reduced the uptake of F′-GpYEEI (Figure 5). Furthermore, the cellular uptake of F′-GpYEEI mediated by C_20_-(HR)_4_ proved to be a concentration-dependent. For example, C_20_-(HR)_4_ increased the uptake of F′-GpYEEI by about a factor of two at 5 µM and increased by a factor of 2.5 at concentration 10 µM as shown in (Figure 6). 

**Figure 7:**
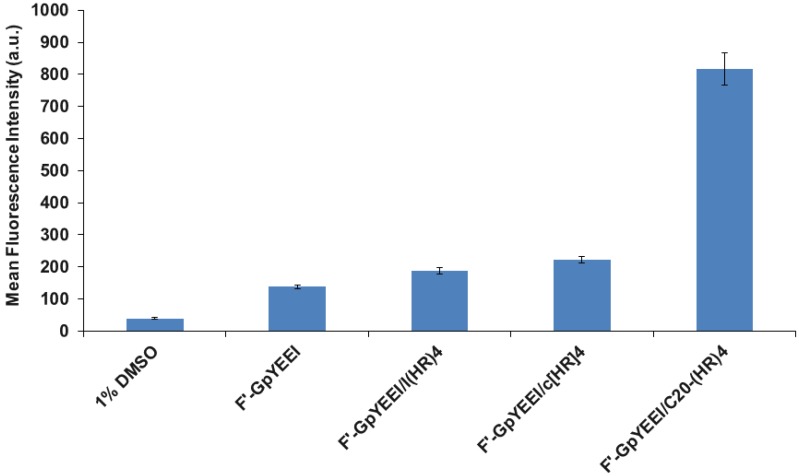
Comparative study for the cellular uptake of F′-GpYEEI (5 µM) by l(HR)_4,_ c[HR]_4_, and C_20_-(HR)_4_ at 50 µM in SK-OV-3 cell line.

Then we compared the cellular uptake of F′-GpYEEI with C_20_-(HR)_4_, l(HR)_4_, and c[HR]_4_ at 50 µM in the SK-OV-3 cells. Figure 7 showed that the C_20_-(HR)_4_ was found to be the most effective in mediating the cellular uptake of F′-GpYEEI after 4 h by more than five folds as compared to ~2 folds by the c[HR]_4_ in the SK-OV-3 cells. 

**Figure 8:**
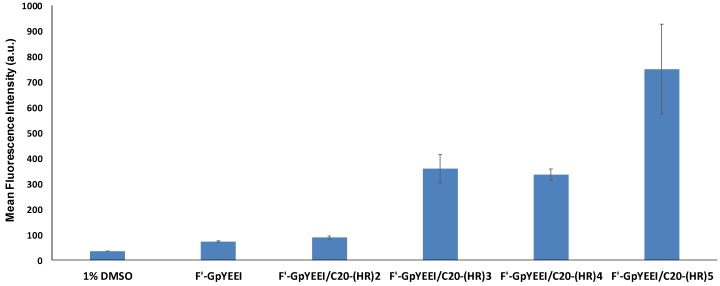
The cellular uptake of F′-GpYEEI at 5 µM concentration by C_20_-(HR)_n_ peptides at 50 µM peptide concentration in CCRF-CEM cell line.

Additionally, the effect of the number of amino acids on the transporting efficacy of C_20_-(HR)_n_ peptides was studied using CCRF-CEM cell line. Increasing the number of histidine and arginine amino acids (n = 2–5), increased the potentiality of C_20_-(HR)_n_ peptides in translocating F′-GpYEEI into the CCRF-CEM cells after 3 h. The C_20_-(HR)_2_ peptide did not mediate the cellular uptake of F′-GpYEEI. C_20_-(HR)_5_ peptide increased the cellular uptake by more than 10 times. Meanwhile, both C_20_-(HR)_4_ and C_20_-(HR)_3_ peptides displayed the same ability in translocating F′-GpYEEI into the CCRF-CEM cells. Both C_20_-(HR)_4_ and C_20_-(HR)_3_ fatty acyl peptides increased the cellular uptake of phosphopeptide by about 4.8 and 4.5 times respectively as compared to the free F′-GpYEEI (Figure 8). 

#### 2.2.3. Anticancer Drug Delivery Using C_20_-(HR)_4_

**Figure 9:**
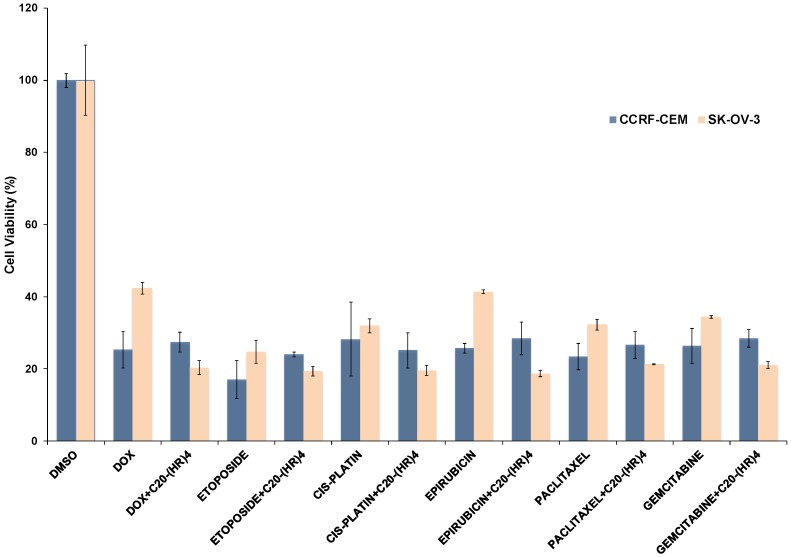
Anti-proliferative assay results for C_20_-(HR)_4_ with different anticancer drugs.

The efficiency of C_20_-(HR)_4_ as drug carrier was evaluated by incubating the peptide at 50 µM concentration with different anticancer drugs including (doxorubicin, etoposide, cisplatin, epirubicin, paclitaxel, and gemcitabine) at a drug concentration of 5 µM for 72 h, as shown in the (Figure 9). The anti-proliferation potency of doxorubicin and epirubicin significantly improved by two fold upon their loading with C_20_-(HR)_4_. The physical mixture of paclitaxel or gemcitabine with C_20_-(HR)_4_ peptide increased the drug toxicity only by 1.5 fold. On the other hand, C_20_-(HR)_4_ did not improve the anti-proliferating potency of etoposide and paclitaxel in CCRF-CEM cell line but the cell viability in SK-OV-3 cell line was reduced by almost one and half fold as compared to the parent analogs.

## 3. Materials and Methods 

### 3.1. General Information

All amino acids were purchased from Chem-Impex International, Inc. All other chemicals and reagents were purchased from Sigma-Aldrich Chemical Co. (Milwaukee, WI, USA). The chemical structures of final products were confirmed by high-resolution MALDI ABX SCIEX TOF/TOF 5800 at the University of California, Irvine, mass spectroscopy facility. The reactions for peptide synthesis were carried out in Bio-Rad polypropylene columns by shaking and mixing under nitrogen using a Glass-Col small tube rotator at room temperature according to our previously reported procedure [20,21,22,23]. The peptides were synthesized by solid-phase synthesis using *N*-(9-fluorenyl)-methoxycarbonyl (Fmoc)-based chemistry and employing Fmoc-L-amino acid building blocks. Linear peptides, (HR)_4_, (HR)_5_ and cyclic peptides, [HR]_4_ and [HR]_5_, were synthesized by solid-phase synthesis using NH_2_-Arg(Pbf)-2-chlorotrityl resin. 2-(1H−Benzotriazole-1-yl)-1,1,3,3-tetramethyluronium hexafluoro phosphate (HBTU) and *N*,*N*-diisopropylethylamine (DIPEA) in *N*,*N*-dimethylformamide (DMF) were used as coupling and activating reagents, respectively. Fmoc deprotection at each step was carried out using piperidine in DMF (20% *v*/*v*). For the peptide synthesis, as representative examples, the synthesis of linear (HR)_4_, cyclic [HR]_4_ and fatty acyl lauryl-(HR)_4_ derivatives are described below.

#### 3.1.1. Synthesis of Linear (HR)_4_

The linear peptide containing eight amino acids of alternative histidine and arginine residues (HRHRHRHR) was synthesized by Fmoc/tBu solid-phase peptide synthesis. H-Arg(Pbf)-2-chlorotrityl resin (0.3 mmol, 732 mg, 0.41 mmol/g) was swelled in DMF and Fmoc groups were deprotected using 20% piperidine/DMF under nitrogen for two times (20 min × 2). Fmoc-His(Trt)-OH (557.74 mg, 0.9 mmol) and Fmoc-Arg(Pbf)-OH (583.89 mg, 0.9 mmol) were coupled alternatively to NH_2_-Arg(Pbf)-2-chlorotrityl resin in the presence of HBTU (341.32. mg, 0.9 mmol) and DIPEA (315 µL, 1.8 mmol) in DMF. Coupling and deprotection cycles were repeated to assemble the sequence of the linear protected peptide. Then, the side-chain deprotection and cleavage from the resin were carried out by a cleavage cocktail reagent “R” (TFA/thioanisole/anisole/1,2-ethanedithiol (EDT), 90:5:2:3, *v*:*v*:*v*:*v*, 15 mL) for 5 h. The crude peptide was precipitated by the addition of cold diethyl ether (75 mL, Et_2_O) and purified by reversed-phase Hitachi HPLC (L-2455) on a water X Bridge TM BEH130 Prep C18 OBD 10 μm ODS reversed-phase column (2.1 cm × 25 cm) using a gradient system. The crude peptide was purified at a flow rate of 10.0 mL/min using a gradient of 0–100% acetonitrile (0.1% TFA) and water (0.1%TFA) over 60 min on RP-HPLC and then was lyophilized to obtain the linear peptide. l(HR)_4_: MALDI-TOF (*m*/*z*): C_48_H_78_N_28_O_9_, calcd. 1190.6507; found 1191.5143 [M]^+^. Similarly, l(HR)_5_ was synthesized. l(HR)_5_: MALDI-TOF (*m*/*z*): C_60_H_97_N_35_O_11_; calcd. 1483.8107; found 1484.7310 [M + H]^+^.

#### 3.1.2. Synthesis of Cyclic [HR]_4_ Peptide

The linear peptide was assembled on H-Arg(Pbf)-2-chlorotrityl resin (0.3 mmol, 732 mg, 0.41 mmol/g) after swelling in DMF for 30 min under nitrogen. Fmoc-His(Boc)-OH (473.9 mg, 0.9 mmol) and Fmoc-Arg(Pbf)-OH (583.9 mg, 0.9 mmol) were coupled alternatively as described above to synthesize linear peptide assembled on the resin. The side chain protected peptide was cleaved from the resin by shaking the resin with a mixture of trifluoroethanol (TFE)/acetic acid/dichloromethane (DCM) (2:2:6, *v*/*v*/*v*, 50 mL) for 2 h. The resin was filtered off and the solution was evaporated to dryness under reduced pressure to yield side-chain protected linear peptide. Then cyclization of the linear peptide was carried out in the presence of DIC (140 μL, 0.9 mmol) and HOAt (122.5 mg, 0.9 mmol) in dry DMF/DCM (200 mL, 3:1 *v*/*v*) under nitrogen stirring for 12 h. After cyclization, the solvent was evaporated and the side chain deprotection was performed by addition of TFA/thioanisole/anisole/EDT (90:5:2:3, *v*:*v*:*v*:*v*, 15 mL) and shaking on a shaker for 5 h. The crude peptide was precipitated by the addition of cold diethyl ether (75 mL, Et_2_O) and purified by reversed-phase Hitachi HPLC (L-2455) on a Waters XBridgeTM BEH130 Prep C18 OBDTM 10 μm ODS reversed-phase column (2.1 cm × 25 cm) using a gradient system. The crude peptide was purified at a flow rate of 10.0 mL/min using a gradient of 0–100% acetonitrile (0.1% TFA) and water (0.1% TFA) over 60 min and then was lyophilized to yield the pure cyclic peptide c[HR]_4_: MALDI-TOF (*m*/*z*) for C_48_H_76_N_28_O_8_, calcd. 1172.6401; found 1173.6167, [M + H]^+^. Similarly, c[HR]_5_ was synthesized. c[HR]_5_: MALDI-TOF (*m*/*z*): C_60_H_95_N_35_O_10_; calcd. 1465.8001; found 1466.8918 [M + H]^+^. 

#### 3.1.3. Synthesis of Lauryl-(HR)_4_ Peptide 

The synthesis of fatty acyl derivatives of linear (HR)_4_ peptides were carried on solid-phase. The peptide was synthesized using Fmoc-Arg(Pbf)-Wang resin (0.3 mmol, 750.0 mg, 0.40 mmol/g). Once, the linear peptide was assembled after coupling Fmoc-His(Trt)-OH (557.74 mg, 0.9 mmol) and Fmoc-Arg(Pbf)-OH (583.90 mg, 0.9 mmol) alternatively using HBTU (341.32. mg, 0.9 mmol) and DIPEA (315 µL, 1.8 mmol) in DMF for 1 h with Fmoc deprotection in between each coupling cycle using 20% piperidine in DMF. After the last coupling was completed, the resin was washed with DMF (3× 15 mL) followed by *N*-terminal Fmoc deprotection using 20% piperidine in DMF (2 × 10 mL, 10 min each). Then, lauric acid (180 mg, 0.9 mmol) was coupled to the *N*-terminal of the linear (HR)_4_ peptide using HBTU (341.32. mg, 0.9 mmol) and DIPEA (315 µL, 1.8 mmol) in the DMF. The resin was washed with DCM (3 × 25 mL) and methanol (3 × 25 mL) and vacuum dried. Then the resin and the side chain protecting groups were cleaved from the peptidyl resin using cleavage cocktail TFA/thioanisole/EDT/anisole (90:5:3:2, *v*:*v*:*v*:*v*, 15 mL) for 5 h. The crude peptide was precipitated by adding cold diethyl ether (100 mL, Et_2_O) and centrifuged at 4000 rpm for 10 min, followed by decantation to obtain the solid precipitate. The solid material was further washed with cold ether (2 ×100 mL) for 2 times. The peptide was resolubilized in a solvent (CH_3_CN + 0.1% TFA and water + 0.1% TFA). The crude peptide was purified by using the reverse-phase high performance liquid chromatography (RP-HPLC) equipped with a Waters XBridgeTM BEH130 Prep C18 column OBDTM 10 μm (19 mm × 250 mm). A gradient of 0–100% acetonitrile and water in 0.1% TFA (*v*/*v*) with a flow rate at 10.0 mL/min was used for the purification. The peptide powder was obtained after lyophilization of pure HPLC fraction. Other fatty acid conjugates of (HR)_4_ were synthesized in a similar manner: 

CH_3_CO-(HR)_4_: MALDI-TOF (*m*/*z*): C_50_H_80_N_28_O_10_; calcd, 1232.6612; found 1233.5829 [M + H]^+^; COOH-(CH_2_)_6_-CO-(HR)_4_: MALDI-TOF (*m*/*z*): C_56_H_90_N_28_O_12_; calcd, 1346.7293; found 1347.5835 [M + H]^+^; CH_3_-(CH_2_)_10_-CO-(HR)_4_: MALDI-TOF (*m*/*z*): C_60_H_100_N_28_O_10_; calcd, 1372.8177; found 1373.8236 [M + H]^+^; CH_3_-(CH_2_)_12_-CO-(HR)_4_: MALDI-TOF (*m*/*z*): C_62_H_104_N_28_O_10_; calcd, 1400.8490 found 1401.6533 [M + H]^+^; CH_3_-(CH_2_)_14_-CO-(HR)_4_: MALDI-TOF (*m*/*z*): C_64_H_108_N_28_O_10_; calcd, 1428.8803; found 1429.9110 [M + H]^+^; CH_3_-(CH_2_)_7_CH=CH(CH_2_)_7_-CO-(HR)_4_: MALDI-TOF (*m*/*z*): C_66_H_109_N_28_O_10_; calcd, 1454.8960; found 1455.6722 [M + H]^+^; CH_3_-(CH_2_)_18_-CO-(HR)_4_: MALDI-TOF (*m*/*z*): C_68_H_111_N_28_O_10_; calcd, 1484.9429; found 1485.7854 [M + H]^+^; CH_3_-(CH_2_)_18_-CO-(HR)_5_: MALDI-TOF (*m*/*z*): C_80_H_135_N_35_O_12_; calcd, 1778.1029; found 1778.8019 [M]^+^; CH_3_-(CH_2_)_18_-CO-(HR)_3_: MALDI-TOF (*m*/*z*): C_56_H_79_N_21_O_8_; calcd, 1191.7829; found, 1192.7028 [M + H]^+^; CH_3_-(CH_2_)_18_-CO-(HR)_2_: MALDI-TOF (*m*/*z*): C_62_H_104_N_28_O_10_; calcd, 898.6229 found, 899.3463; [M + H]^+^. 

### 3.2. Cell Culture

Human leukemia CCRF-CEM (ATCC no. CCL-119) and ovarian carcinoma SK-OV-3 (ATCC no. HTB-77) cell lines were obtained from American Type Culture Collection. Cells were grown on 75 cm^2^ cell culture flasks with Roswell Park Memorial Institute (RPMI)-1640 medium (for CCRF-CEM) and Eagle’s minimum essential medium (EMEM) (for SK-OV-3), supplemented with 10% fetal bovine serum (FBS) and 1% penicillin−streptomycin solution (10,000 units of penicillin and 10 mg of streptomycin in 0.9% NaCl) in a humidified atmosphere of 5% CO_2_, 95% air at 37 °C. All bioassays were performed in triplicate.

### 3.3. Cell Viability Assays Using MTT

The cytotoxicity assay was performed via an MTS proliferation assay in CCRF-CEM and SK-OV-3 cells. 5000 cells were incubated with 100 µL of complete media and allowed to grow for overnight in each well of the 96 well plates. The linear and cyclic peptides at concentrations (5, 10, 25, 50 and 100 µM) were added to the cells and incubated at 37 °C with 5% carbon dioxide for 72 h. After 72 h of incubation, the pH of media were tested. Both cell lines with/without (HR)_4_ treatment were found to be acidic (~6.5) whereas the cells treated with C_20_-(HR)_4_ were slightly basic (pH ~ 7.5). Then 20 µL of MTS reagent was added to each well. CCRF-CEM cells were incubated for 3 h, and SK-OV-3 cells were incubated for 1 h at 37 °C with 5% carbon dioxide. The fluorescence intensity of the formazan product was measured at 490 nm using a Spectra Max M2 microplate spectrophotometer. The percentage of cell survival was calculated as [(OD value of cells treated with the test mixture of compounds) − (OD value of culture medium)]/[(OD value of control cells) − (OD value of culture medium)] × 100%.

### 3.4. Cellular Uptake Studies of Fluorescein-Labeled Phosphopeptide by Flow Cytometry

The cellular uptake assays were performed via FACS analysis using SK-OV-3 and CCRF-CEM cells in 6-well plates. The fluorescein-labeled phosphopeptide (F′-GpYEEI) was added to the well plates at a concentration of 5 µM, and the peptides were added to the well in each plate at a concentration of 5, 10 and 50 µM. The cells were incubated at 37 °C with 5% carbon dioxide for 2 h. The cells were digested with 0.25% trypsin, 0.53 mM EDTA for 5 min to detach from the surface followed by addition of 2 mL of complete media to deactivate the trypsin. The cells were centrifuged at 2500 rpm and washed with PBS for two times. Finally, the cells were resuspended in 400 µL of flow cytometry buffer and analyzed by flow cytometry. The data presented are based on the mean fluorescence signal for 10,000 cells collected. All assays were performed in triplicate. 

### 3.5. Antiproliferative Assay of the Anticancer Drugs Using C_20_-(HR)_4_

The antiproliferative assay was performed using MTT proliferation assay in CCRF-CEM and SK-OV-3 cells. The cells were incubated with 100 µL of complete media overnight in a 96 well plate. The anticancer drugs namely (Doxorubicin, Etoposide, Cis-platin, Epirubicin, Paclitaxel, and Gemcitabine) were added to the well plates at a concentration of 5 µM and C_20_-(HR)_4_ was added to the cells at a concentration of 50 µM. The cells were incubated at 37 °C with 5% CO_2_ for 72 h. Then 20 µL of MTT was added to each well. After addition of MTT reagent, CCRF-CEM cells were incubated for 3 h and SK-OV-3 cells were incubated for 1 h at 37 °C with 5% CO_2_. The absorbance (and thus percent cell viability) was obtained at a wavelength of 490 nm using a microplate reader.

## 4. Conclusions

In conclusion, a new series of linear and cyclic homochiral amphipathic peptides, which consists of alternating arginine and histidine amino acids, were synthesized. Both linear and cyclic peptides showed 0–13% cytotoxicity on SK-OV-3 and CCRF-CEM. Among the investigated peptides, c[HR]_4_ proved to be promising as molecular transporters for F′-GpYEEI phosphopeptide. The attachment of different fatty acids to l(HR)_4_ peptide modulated its toxicity profile and molecular transporter property. C_2_-, C_8_-, C_12_-, C_14_-, C_18_-(HR)_4_ were not toxic up to 50 µM concentration while C_16_-(HR)_4_ and C_20_-(HR)_4_ exhibited a significant reduction in the cell viability at the same concentration. Our studies revealed that the cellular uptake of F′-GpYEEI phosphopeptide by C_20_-(HR)_4_ was significantly improved and was concentration dependent. Increasing the number of amino acids increased the efficacy of the C_20_-[HR]_n_ peptide in translocating F′-GpYEEI phosphopeptide into the cells. When C_20_-(HR)_4_ mixed with several anticancer drugs, the antiproliferation potency of doxorubicin and epirubicin increased more than the parent alone in SK-OV-3 cell line. 

## Supplementary Materials

The supplementary materials are available online containing MALDI-MS spectra of synthesized peptides.
