# Supplementary material for: Design, Synthesis, and Evaluation of Homochiral Peptides Containing Arginine and Histidine as Molecular Transporters
Source: Molecules. 2018 Jun 29;23(7):1590. doi: 10.3390/molecules23071590 (PMC6100079; doi:10.3390/molecules23071590)

Design, Synthesis, and Evaluation of Homochiral Peptides Containing Arginine and Histidine as Molecular Transporters

Naglaa Salem El-Sayed[1,2], Taryn Miyake[1], Amir Nasrolahi Shirazi[1], Shang Eun Park[1], Jimmy Clark[1], Stephani Buchholz[1], Keykavous Parang[1], and Rakesh Tiwari[1,*]

1 Center for Targeted Drug Delivery, Department of Biomedical and Pharmaceutical Sciences, Chapman University School of Pharmacy, Harry and Diane Rinker Health Science Campus, Irvine, California 92618, United States;

2 Cellulose and Paper Department, National Research Center, Dokki 12622, Cairo, Egypt;

Correspondence: tiwari@chapman.edu; Tel.: +1-714-516-5483

Content

- MALDI-MS spectra of synthesized peptides. (Slide #3-16)

1. Linear $(HR)_4$

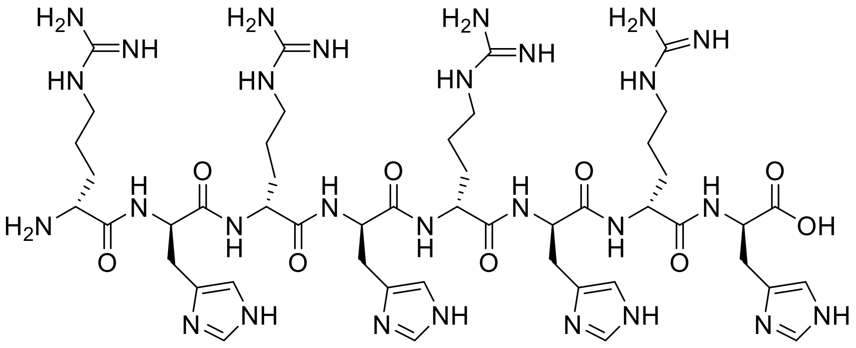

Chemical Formula: $C_{48}H_{78}N_{28}O_9$
Exact Mass: 1190.6507

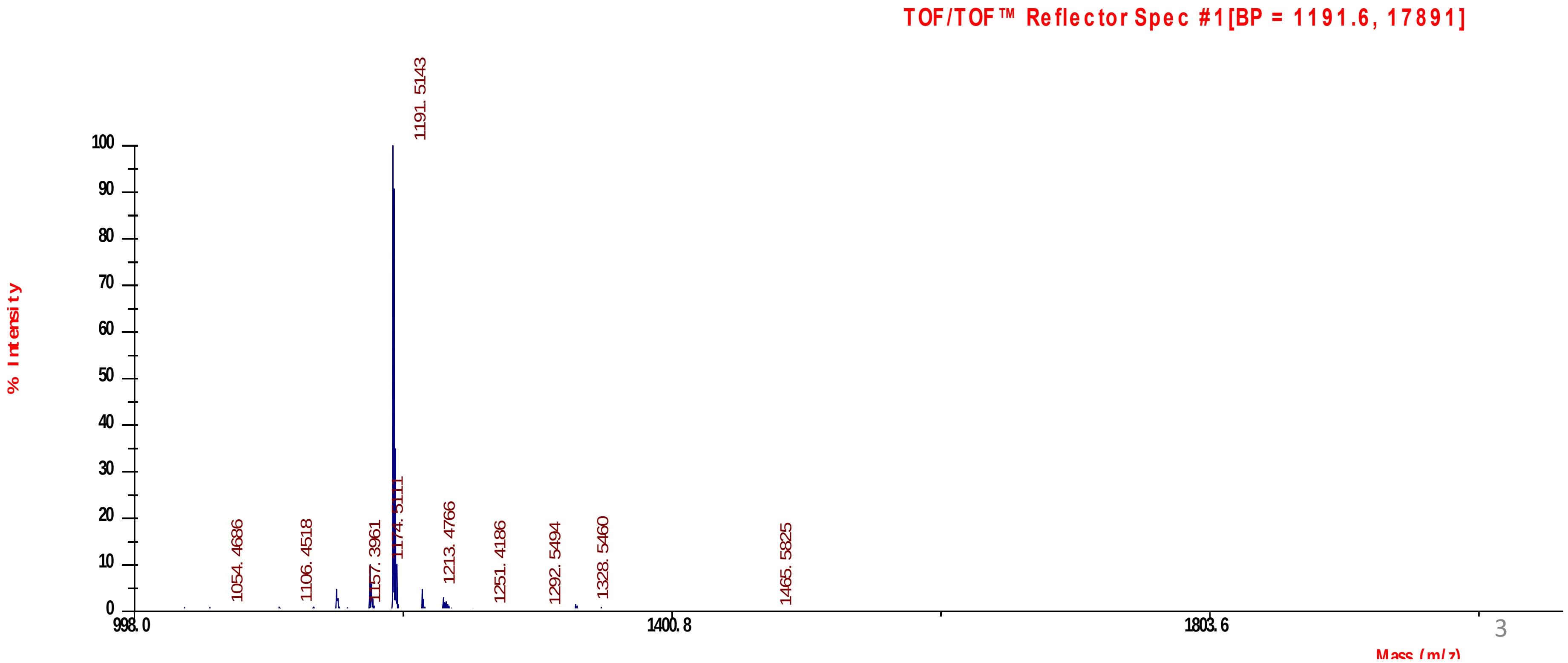

2. Cyclic $[HR]_4$

Chemical Formula: $C_{48}H_{76}N_{28}O_8$

Exact Mass: 1172.6401

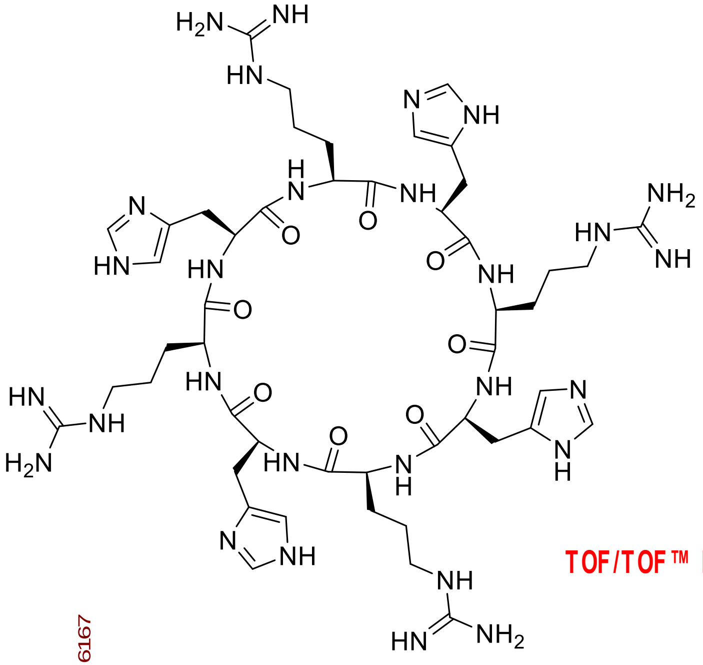

TOF/TOF™ Reflector Spec #1[BP = 1173.6, 63]

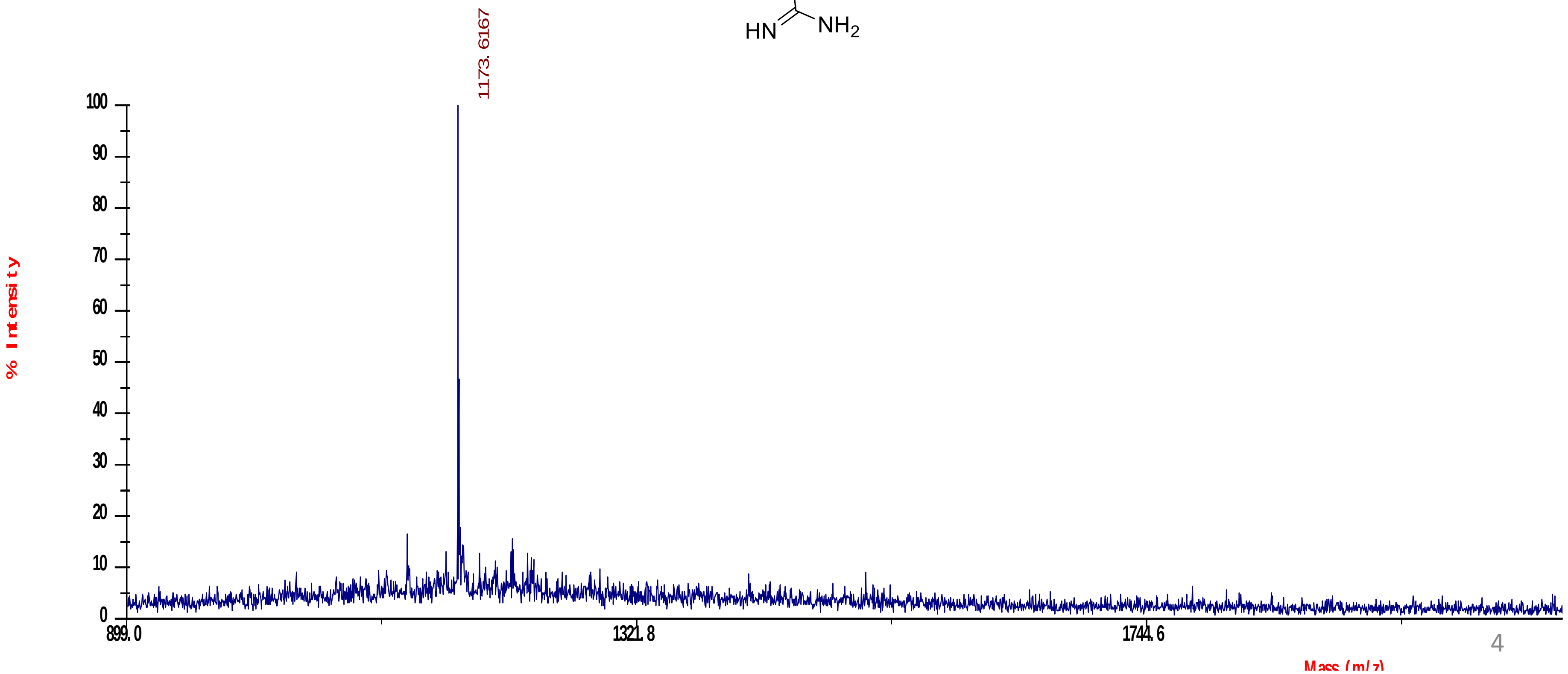

3. Linear $(HR)_5$

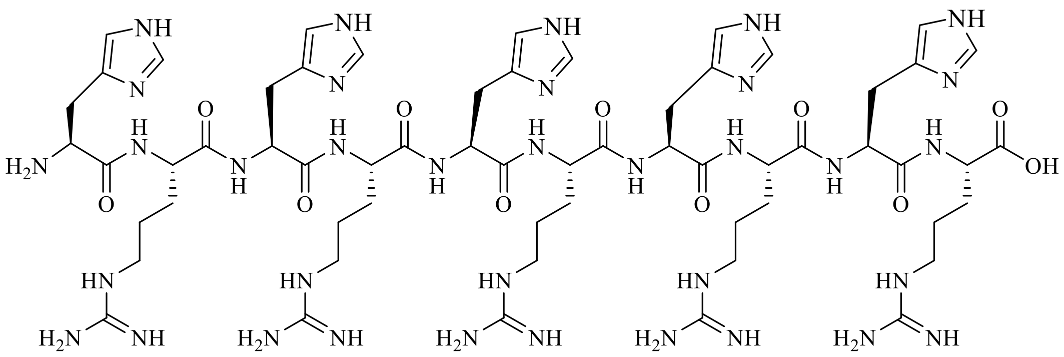

Chemical Formula: $C_{60}H_{97}N_{35}O_{11}$
Exact Mass: 1483.8107

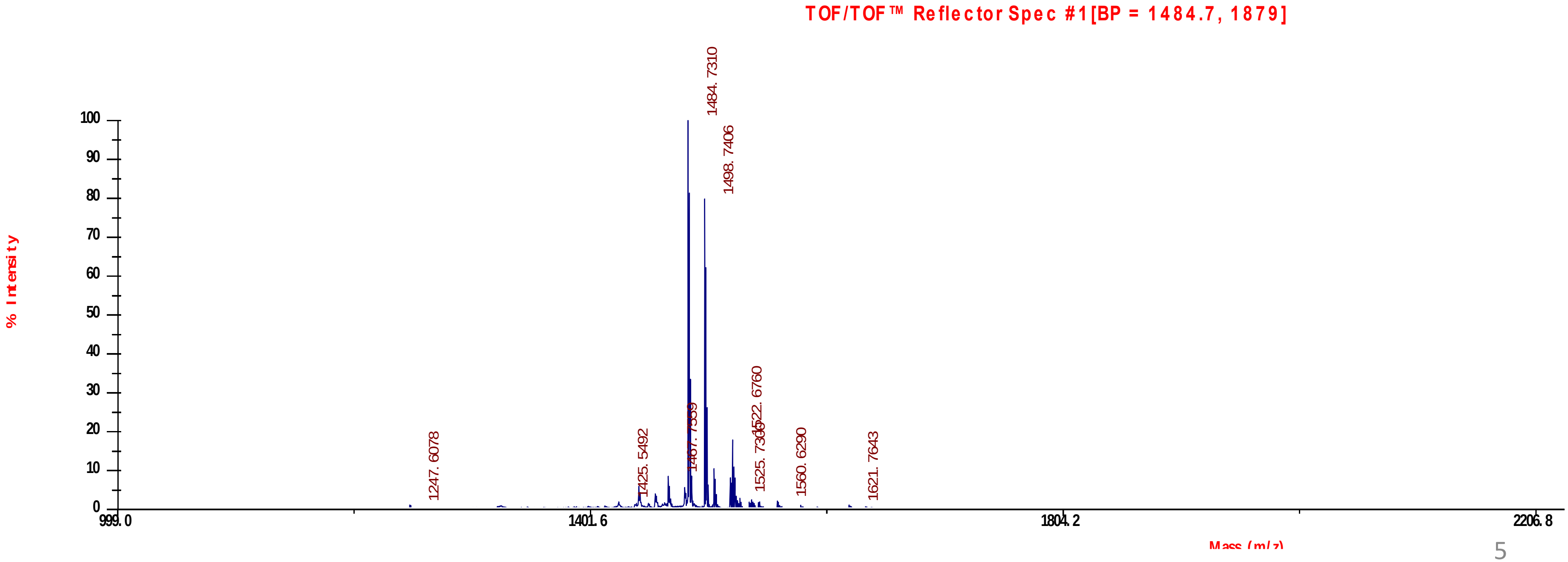

4. Cyclic $[HR]_5$

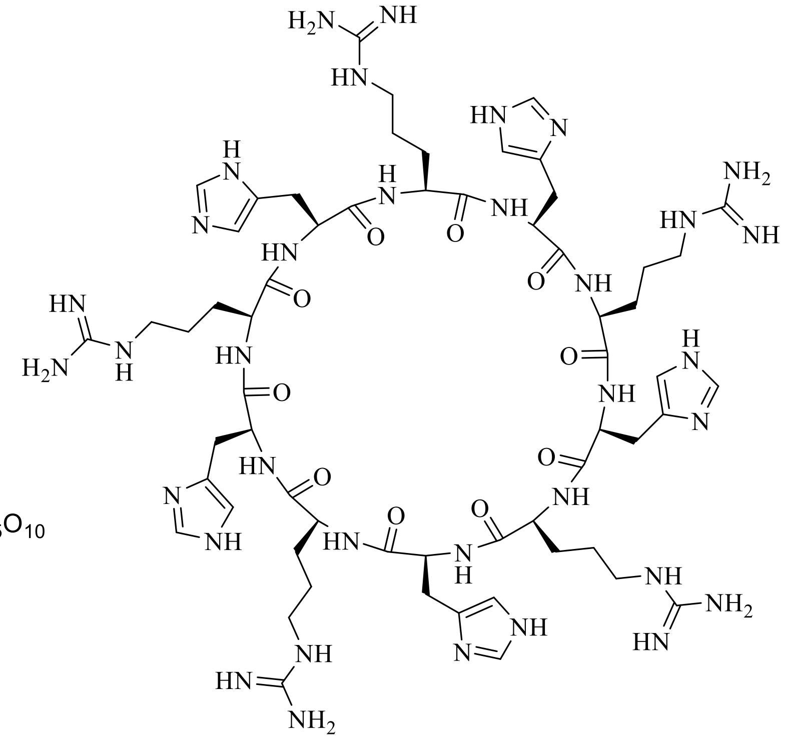

Chemical Formula: $C_{60}H_{95}N_{35}O_{10}$
Exact Mass: 1465.8001

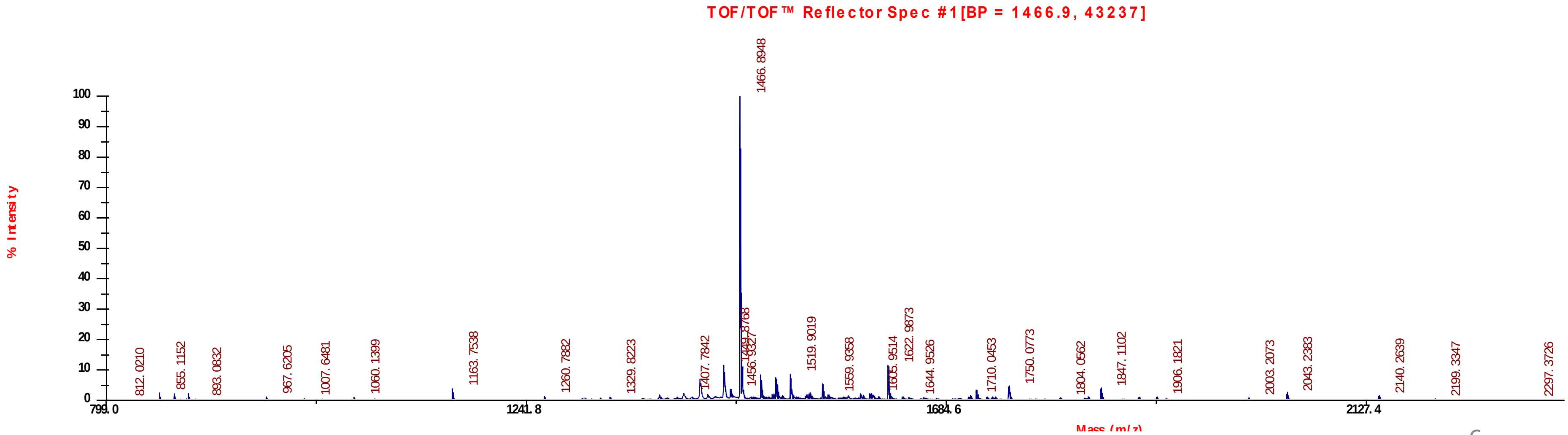

5. C_2-$(HR)_4$

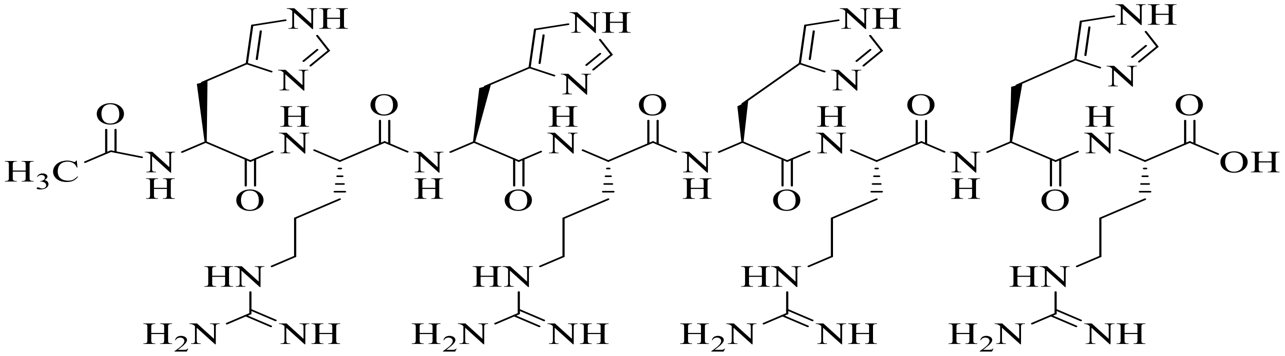

Chemical Formula: $C_{50}H_{80}N_{28}O_{10}$
Exact Mass: 1232.6612

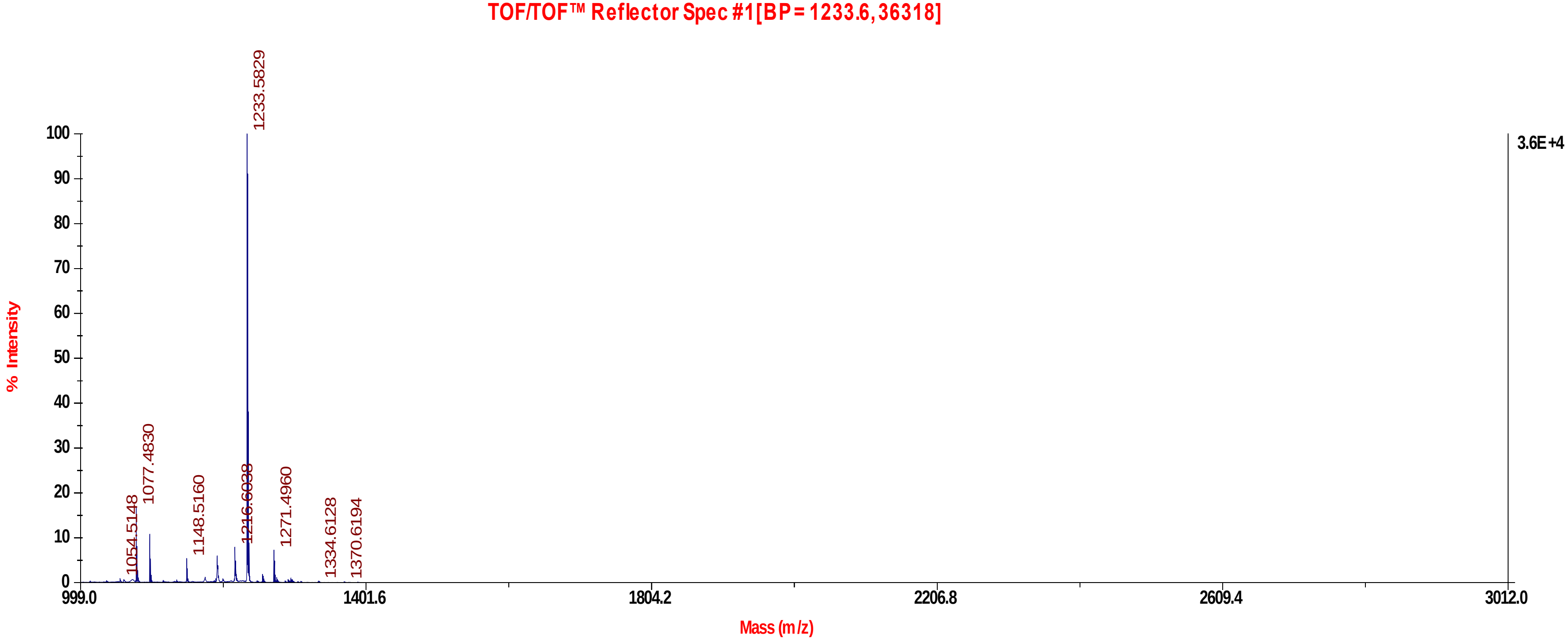

6. C_8-(HR)$_4$

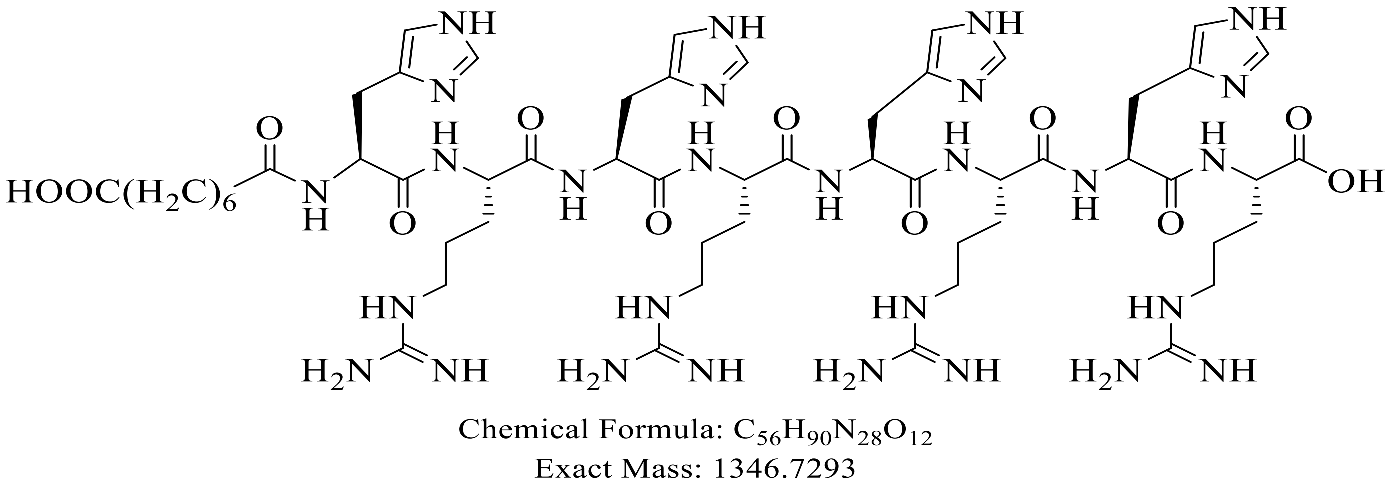
HOOC(H2C)6
Chemical Formula: C56H90N28O12
Exact Mass: 1346.7293

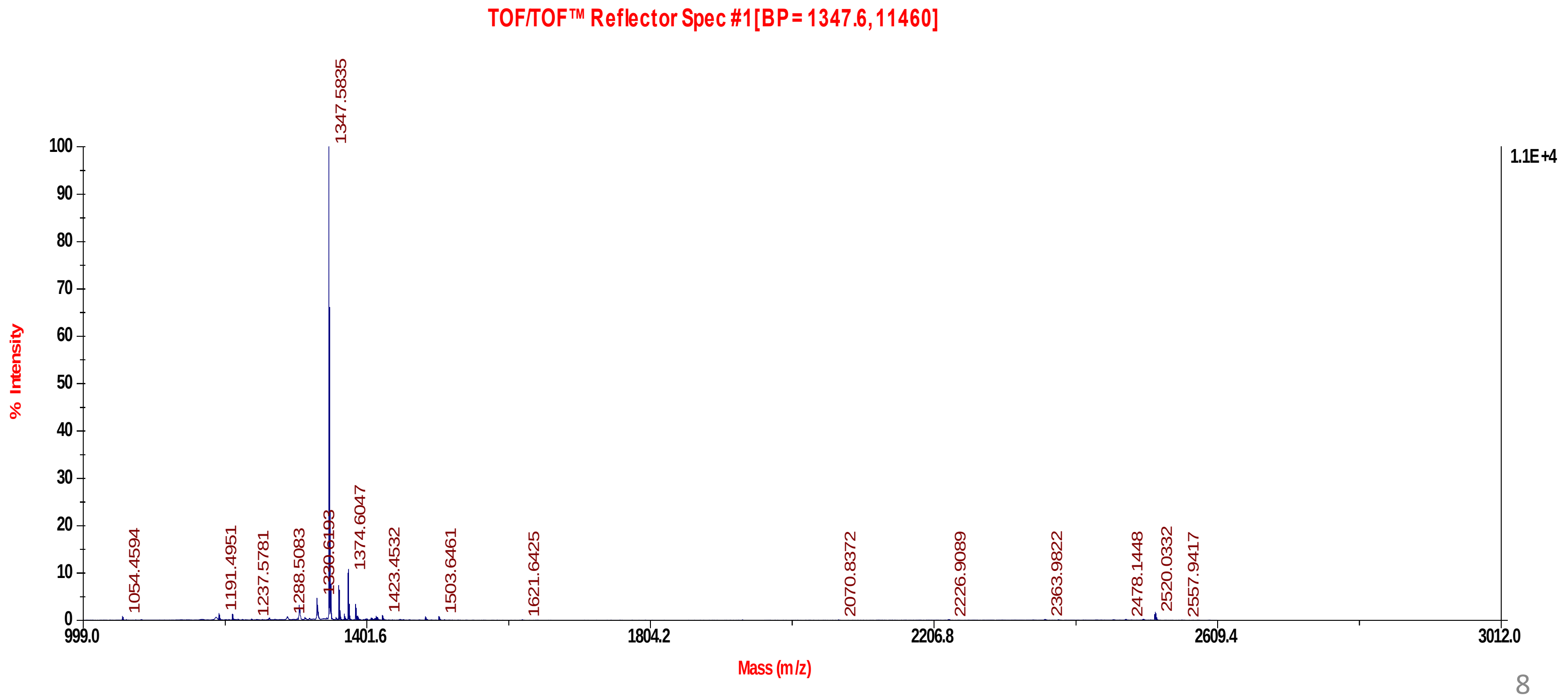
TOF/TOF™ Reflector Spec #1[BP = 1347.6, 11460]
1347.5835
1054.4594
1191.4951
1237.5781
1288.5083
1330.6193
1374.6047
1423.4532
1503.6461
1621.6425
2070.8372
2226.9089
2363.9822
2478.1448
2520.0332
2557.9417
1.1E+4
% Intensity
Mass (m/z)
999.0
1401.6
1804.2
2206.8
2609.4
3012.0

7. C_{12}-(HR)$_4$

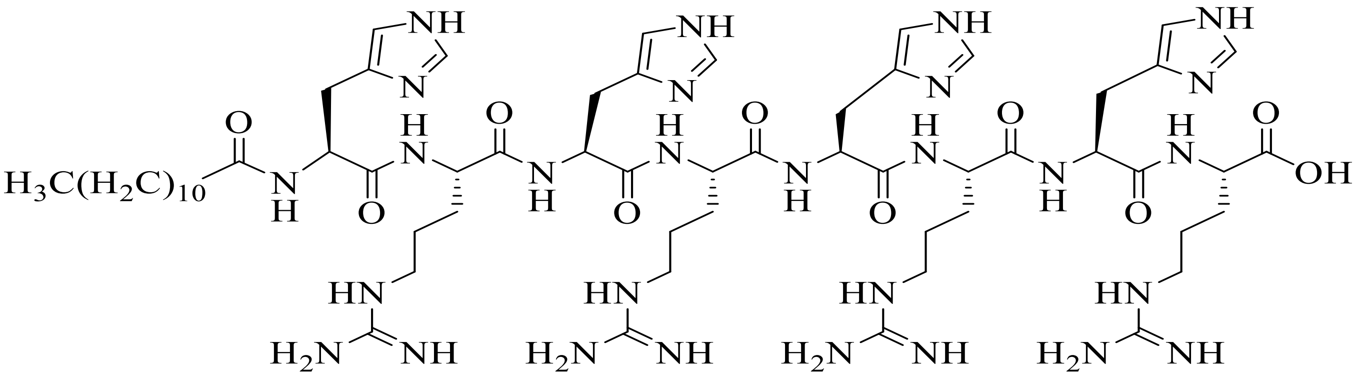

Chemical Formula: $C_{60}H_{100}N_{28}O_{10}$
Exact Mass: 1372.8177

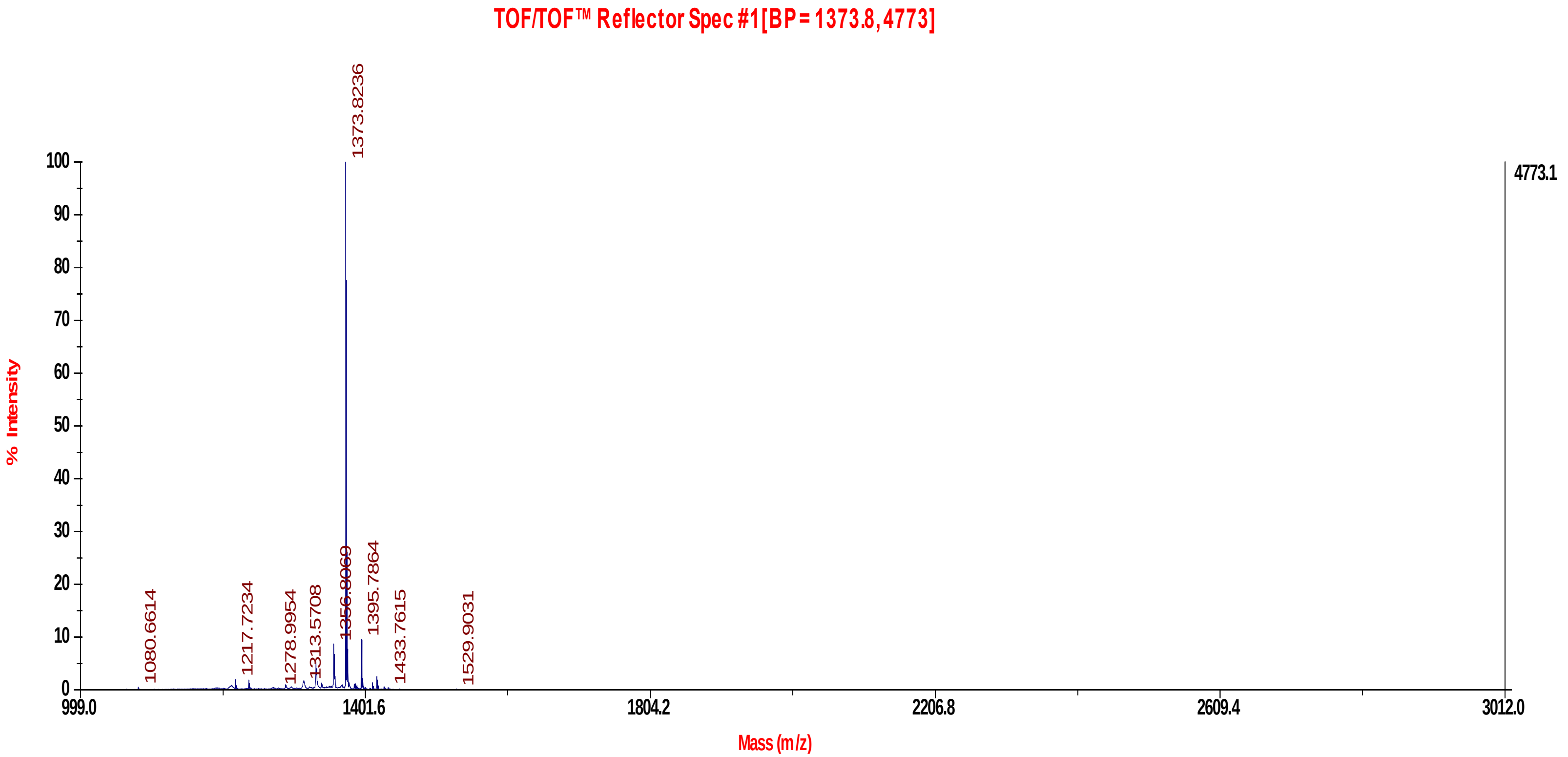

8. C_{14}-(HR)$_4$

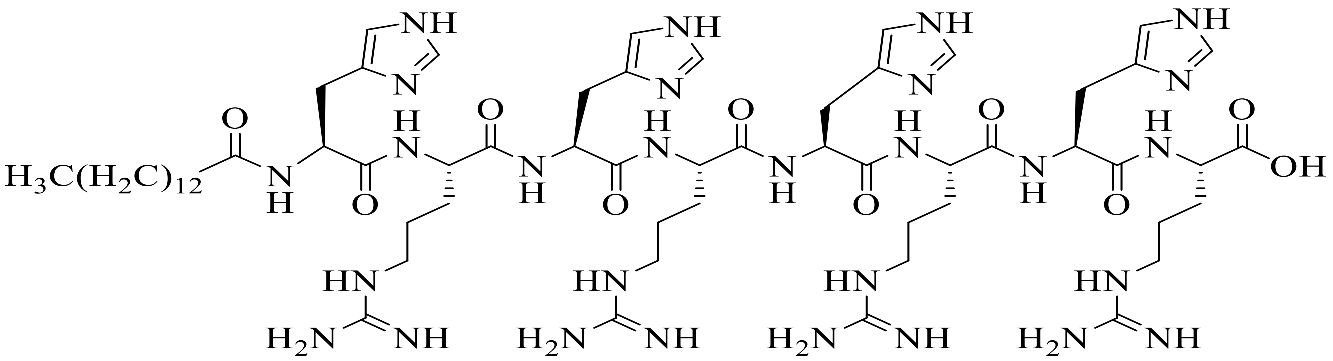

Chemical Formula: $C_{62}H_{104}N_{28}O_{10}$
Exact Mass: 1400.8490

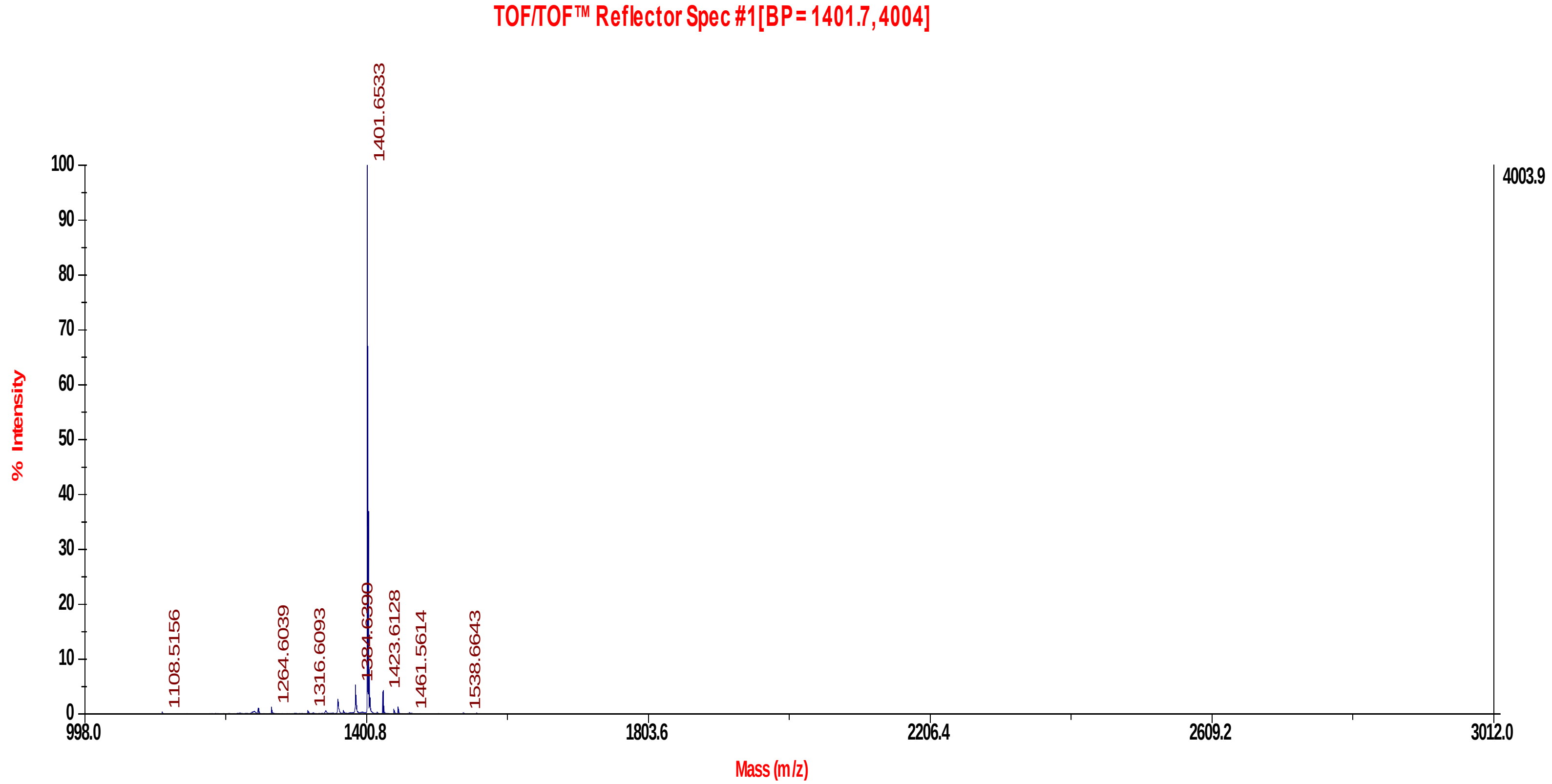

9. C_{16}-$(HR)_4$

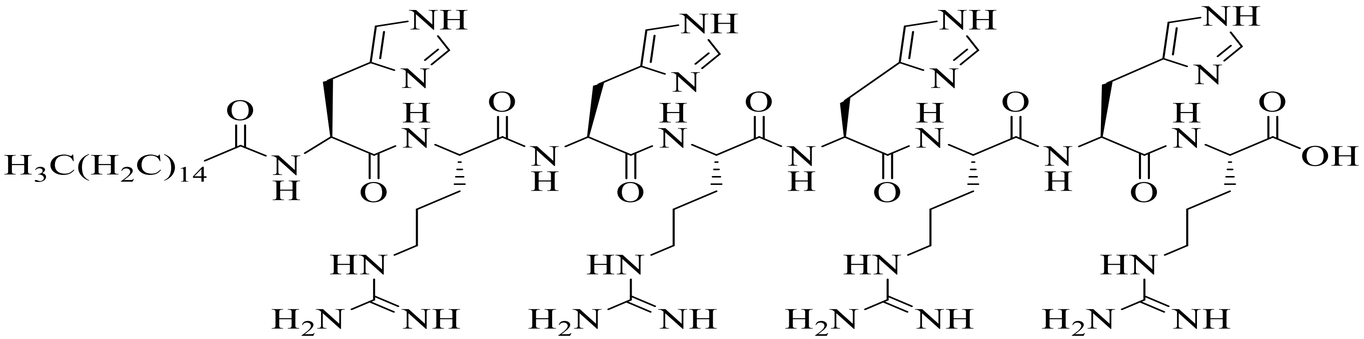

Chemical Formula: $C_{64}H_{108}N_{28}O_{10}$
Exact Mass: 1428.8803

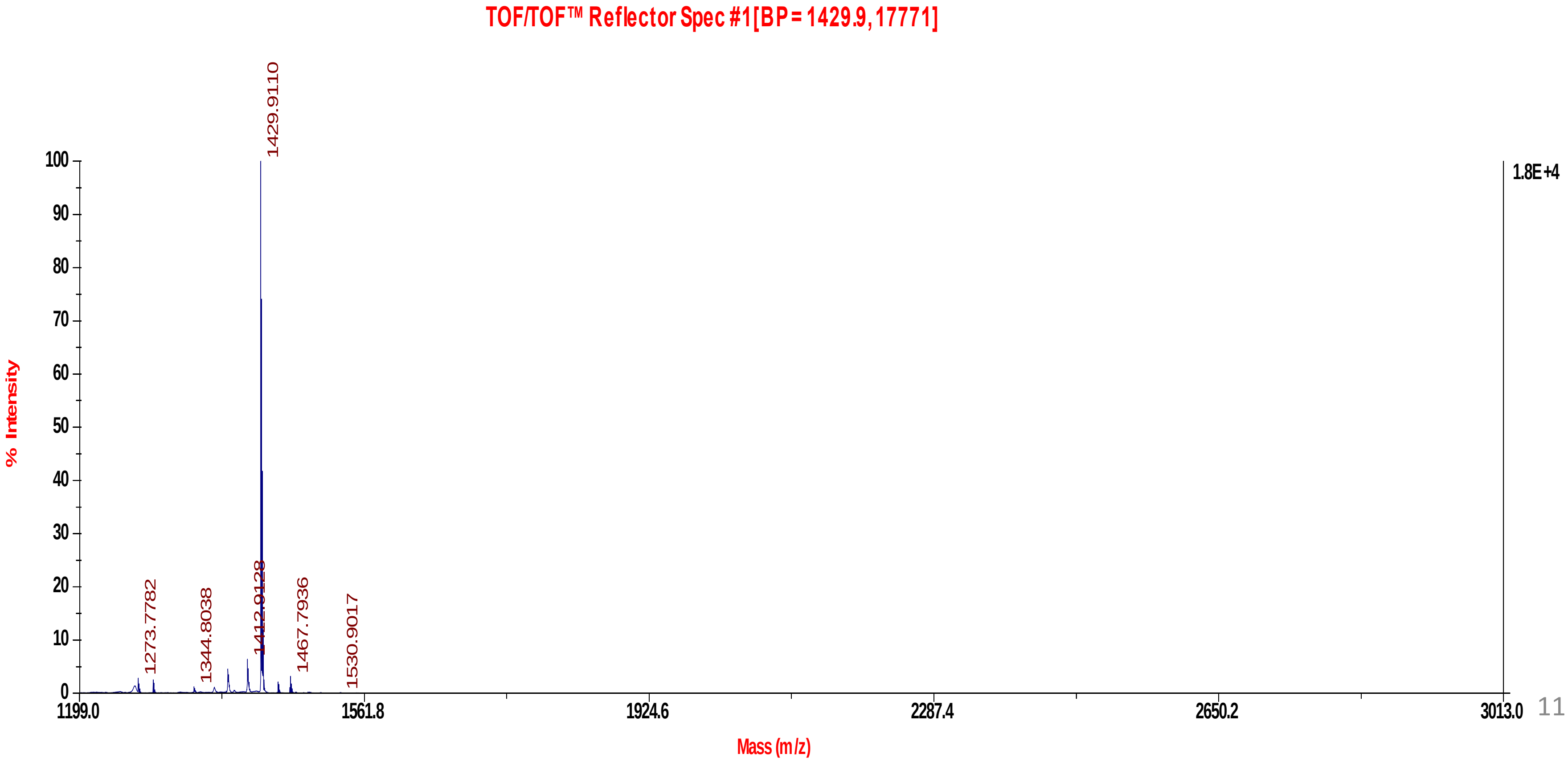

10. C_{18}-$(HR)_4$

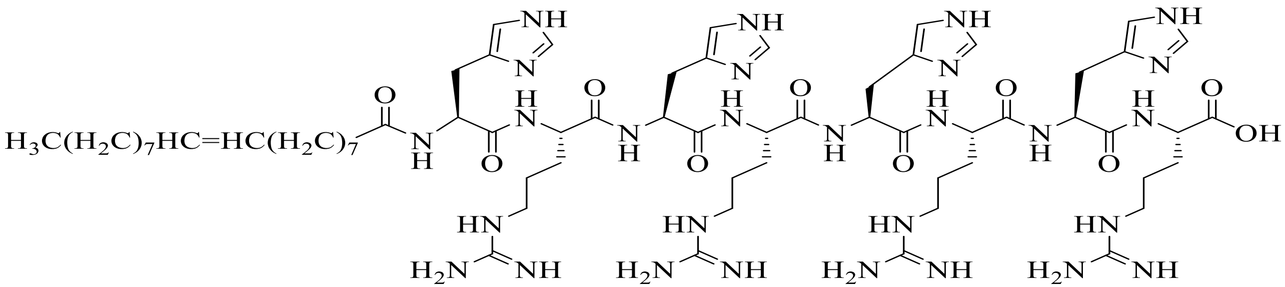

Chemical Formula: $C_{66}H_{110}N_{28}O_{10}$
Exact Mass: 1454.8960

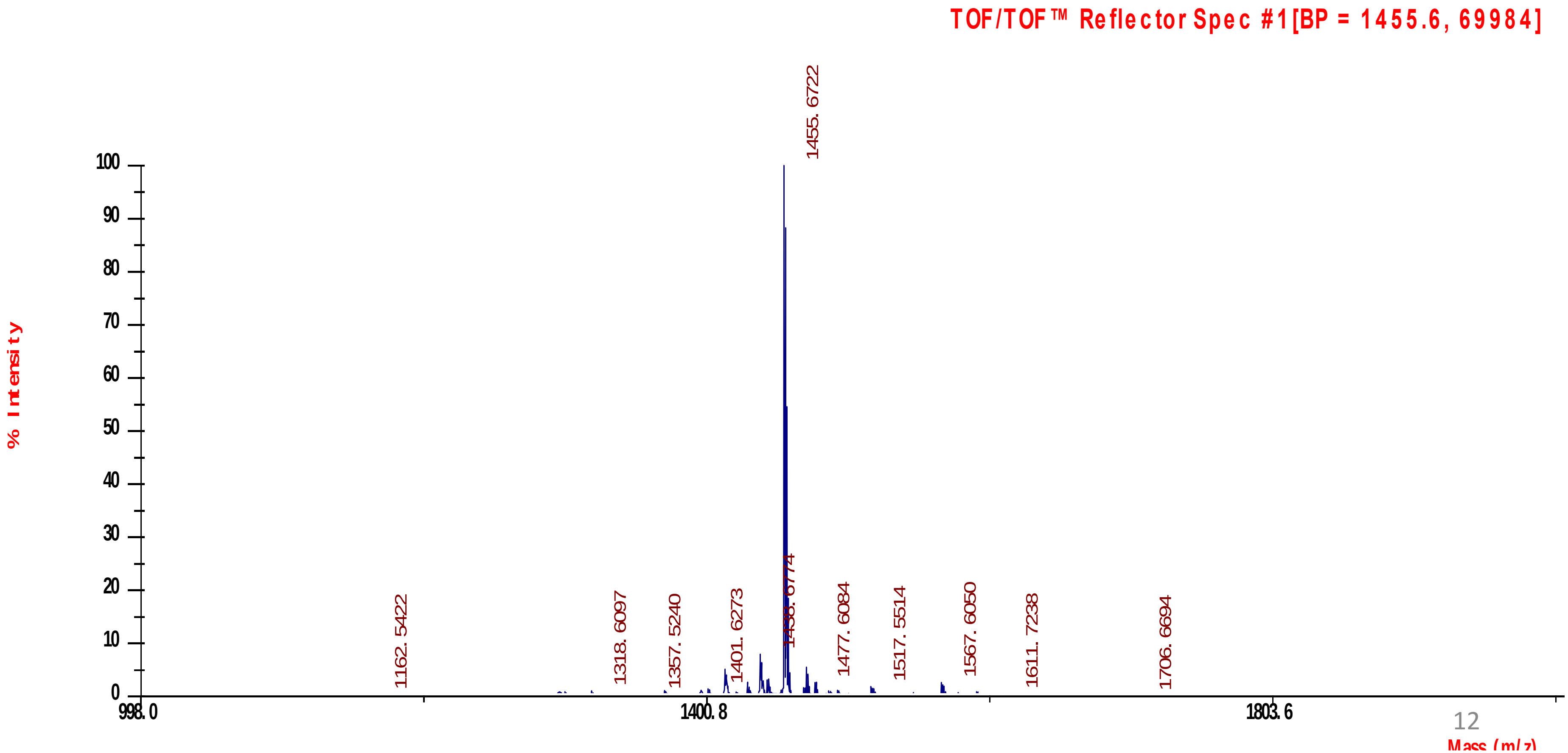

11. C_{20}-$(HR)_4$

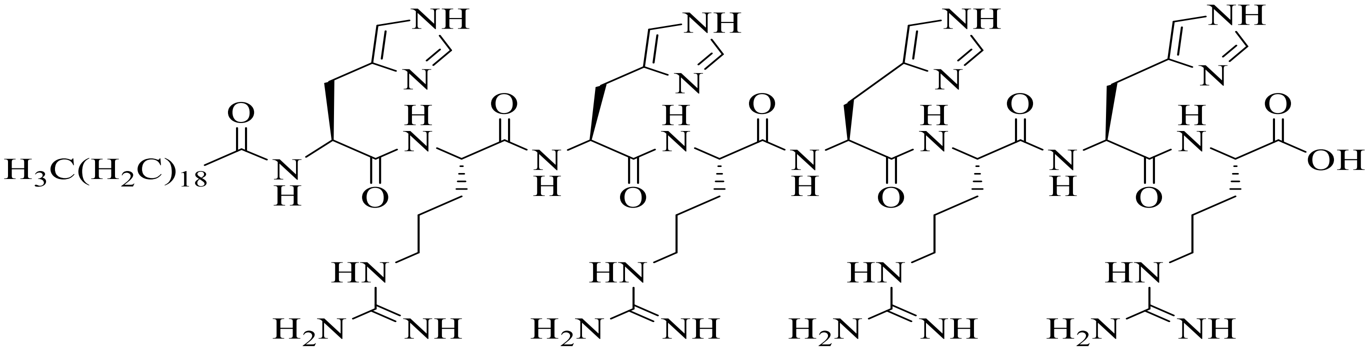

Chemical Formula: $C_{68}H_{116}N_{28}O_{10}$
Exact Mass: 1484.9429

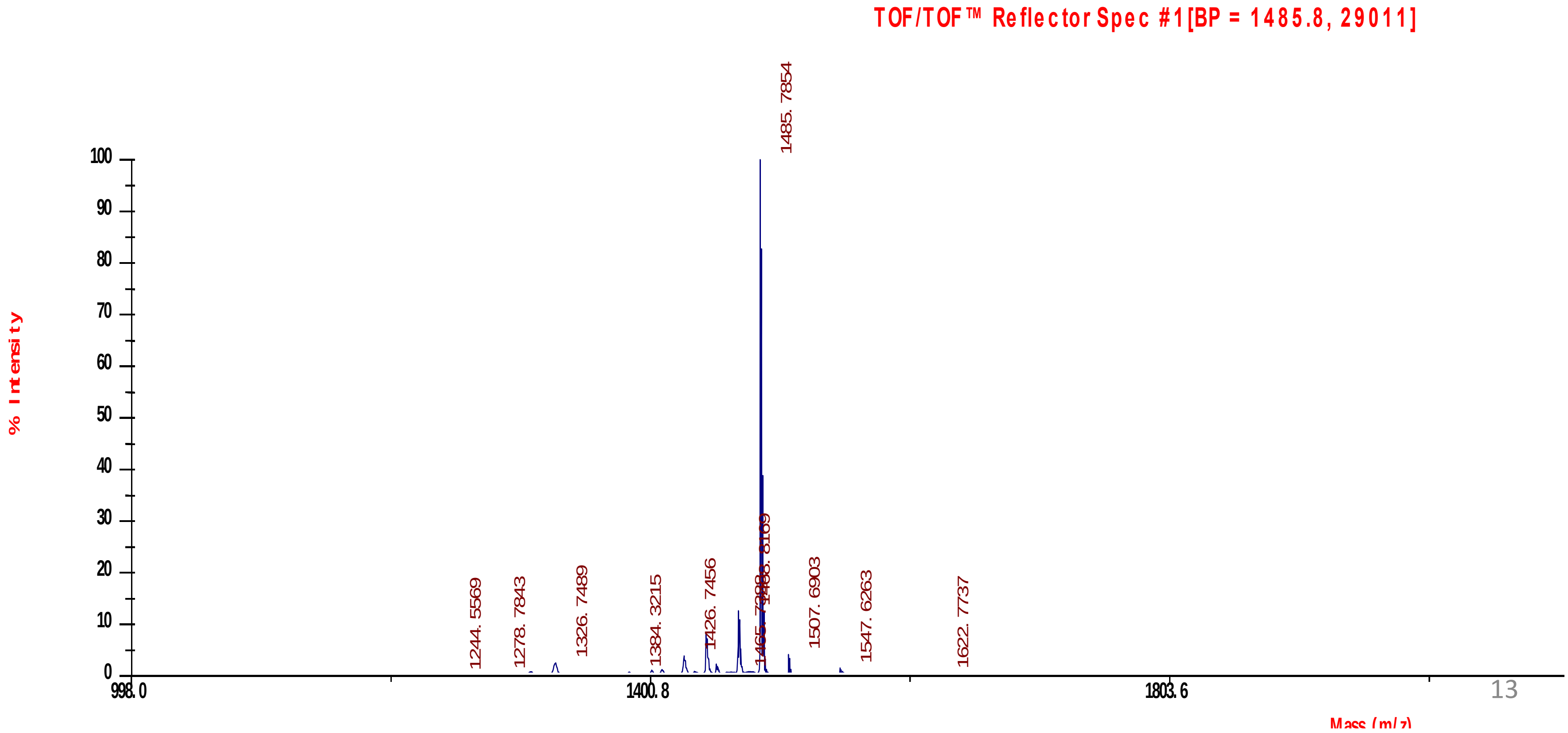

11. C_{20}-(HR)$_2$

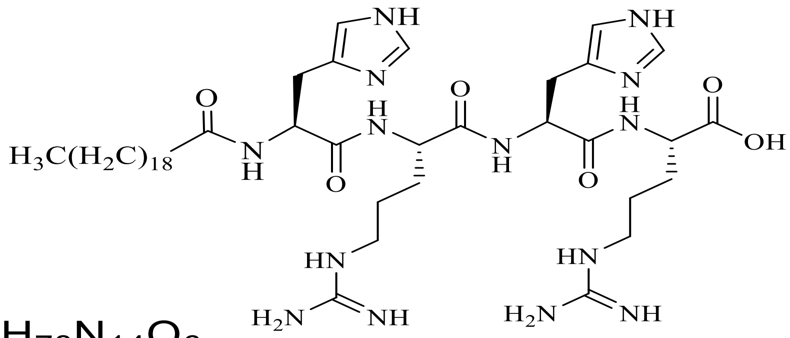

Chemical Formula: $C_{44}H_{78}N_{14}O_6$
Exact Mass: 898.6229

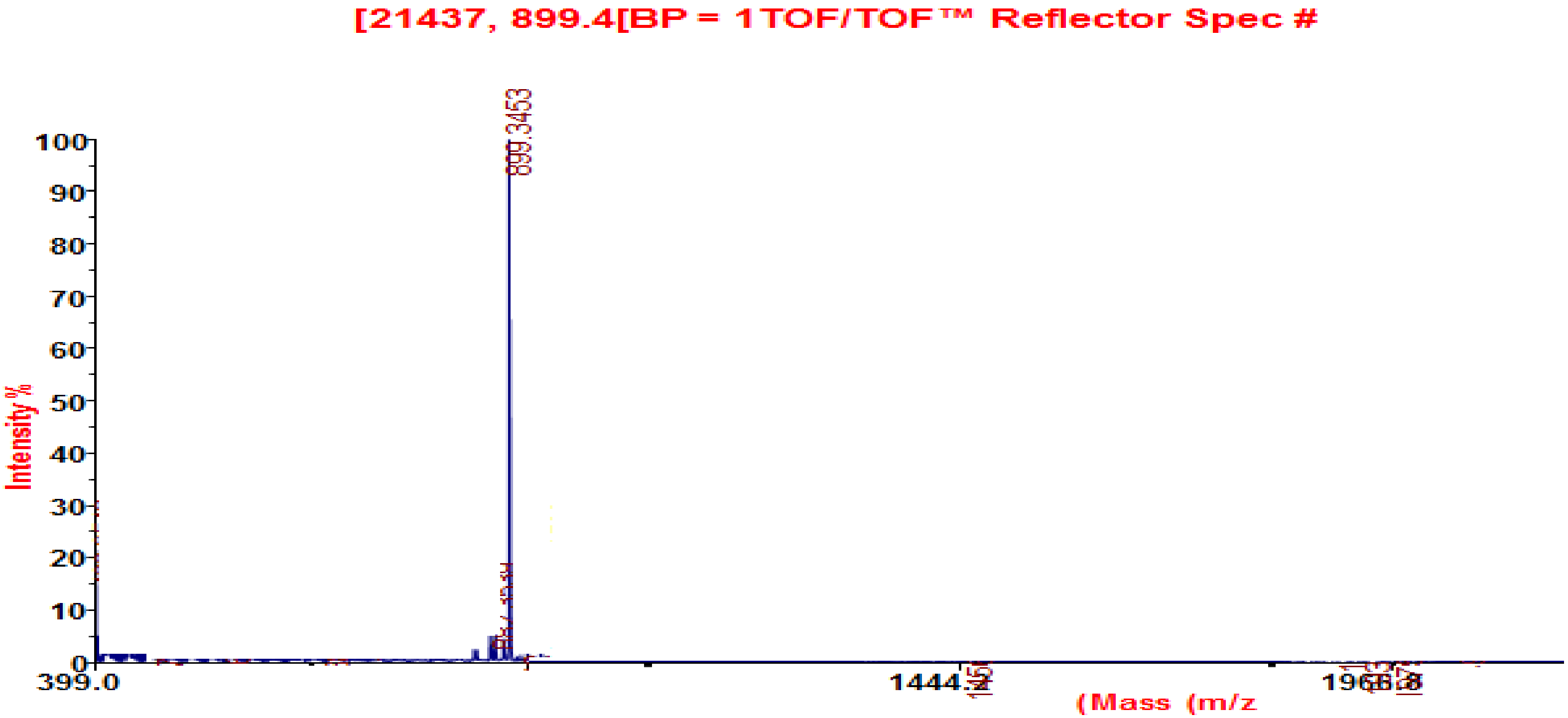

12. C_{20}-$(HR)_3$

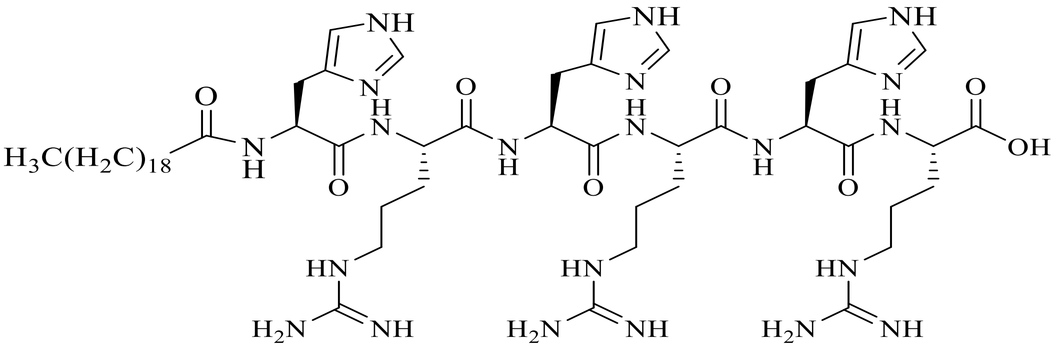

Chemical Formula: $C_{56}H_{97}N_{21}O_8$
Exact Mass: 1191.7829

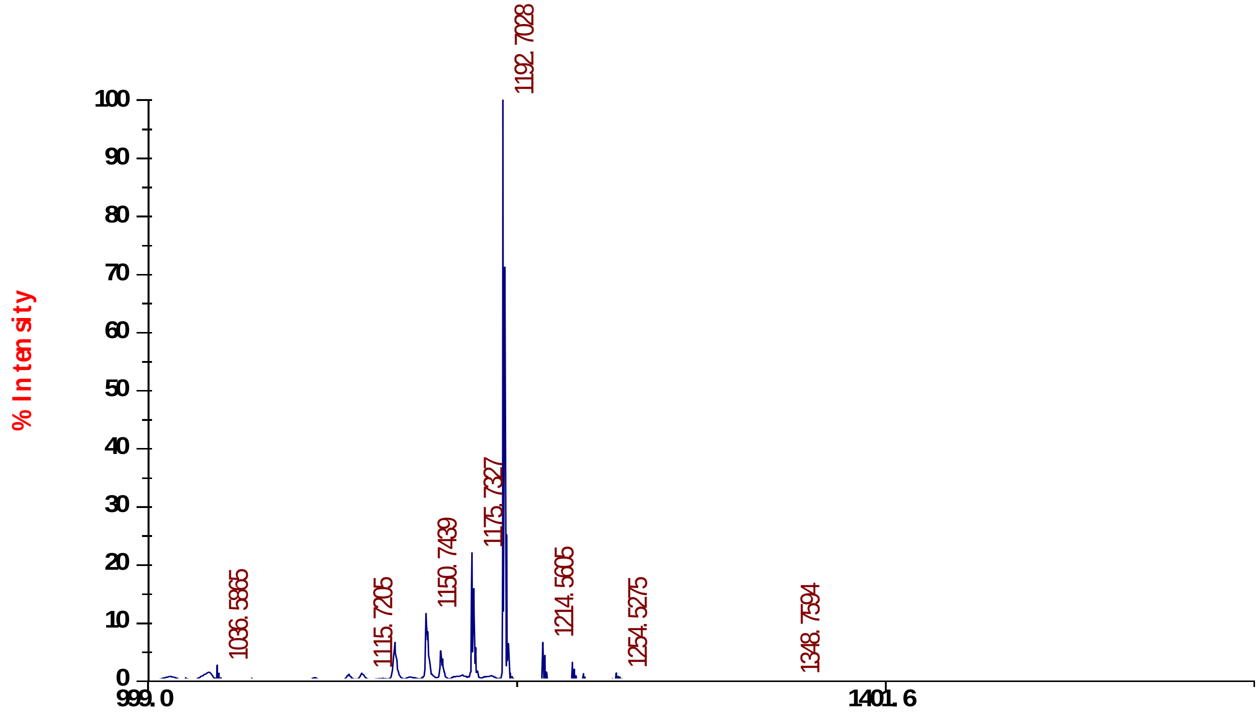

13. C_{20}-$(HR)_5$

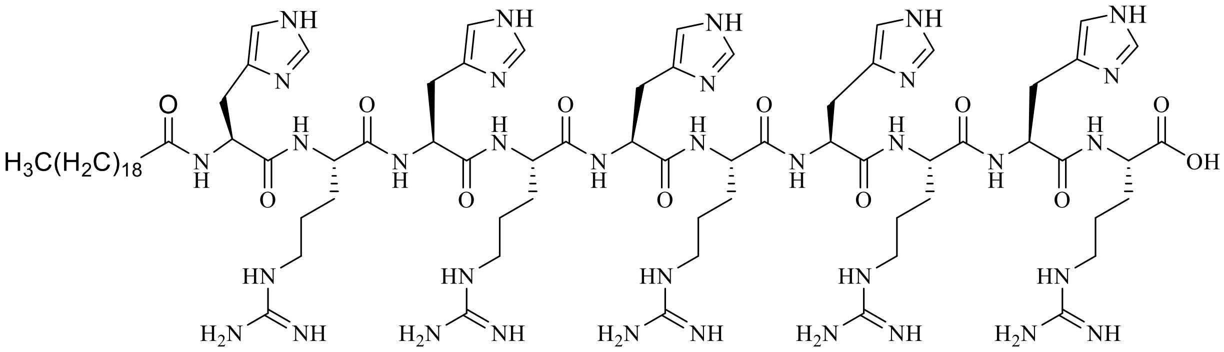

Chemical Formula: $C_{80}H_{135}N_{35}O_{12}$
Exact Mass: 1778.1029

TOF/TOF™ Reflector Spec #1[BP = 1779.8, 5937]

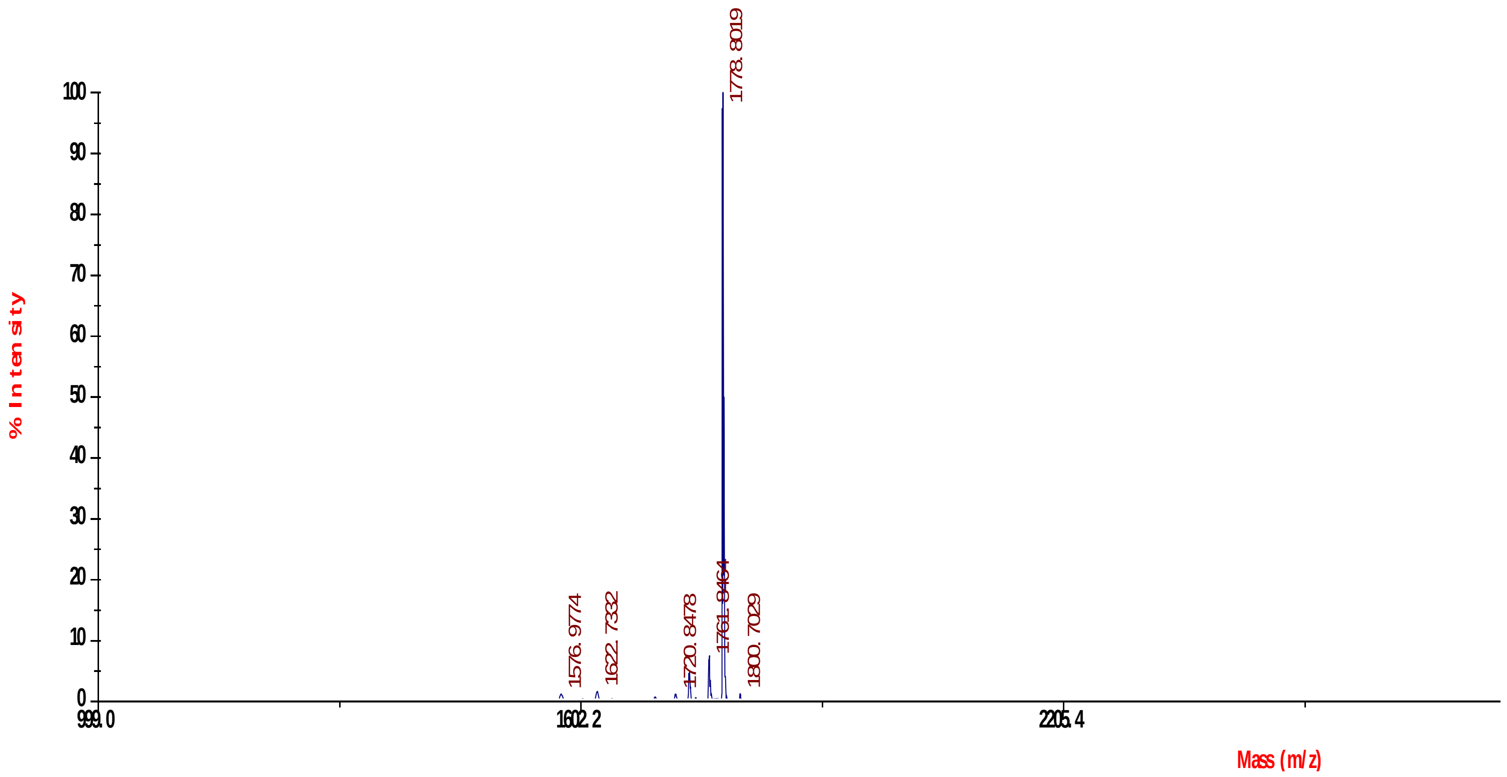

Supplement: Supplementary file 1 [file molecules-23-01590-s001.pdf]
